# Toward a comprehensive evidence map of overview of systematic review methods: paper 2—risk of bias assessment; synthesis, presentation and summary of the findings; and assessment of the certainty of the evidence

**DOI:** 10.1186/s13643-018-0784-8

**Published:** 2018-10-12

**Authors:** Carole Lunny, Sue E. Brennan, Steve McDonald, Joanne E. McKenzie

**Affiliations:** 1 Cochrane Australia, School of Public Health and Preventive Medicine, Monash University, Melbourne, Australia; 2 School of Public Health and Preventive Medicine, Monash University, 553 St Kilda Rd, Melbourne, VIC 3004 Australia

**Keywords:** Overview of systematic reviews, Overview, Meta-review, Umbrella review, Review of reviews, Systematic review methods, Evidence map, Evaluation of methods, Methodology, Assessment of risk of bias in systematic reviews

## Abstract

**Background:**

Overviews of systematic reviews (SRs) attempt to systematically retrieve and summarise the results of multiple systematic reviews. This is the second of two papers from a study aiming to develop a comprehensive evidence map of the methods used in overviews. Our objectives were to (a) develop a framework of methods for conducting, interpreting and reporting overviews (stage I)—the *M*ethods for *O*verviews *o*f *R*eviews (MOoR) framework—and (b) to create an evidence map by mapping studies that have evaluated overview methods to the framework (stage II). In the first paper, we reported findings for the four initial steps of an overview (specification of purpose, objectives and scope; eligibility criteria; search methods; data extraction). In this paper, we report the remaining steps: assessing risk of bias; synthesis, presentation and summary of the findings; and assessing certainty of the evidence arising from the overview.

**Methods:**

In stage I, we identified cross-sectional studies, guidance documents and commentaries that described methods proposed for, or used in, overviews. Based on these studies, we developed a framework of possible methods for overviews, categorised by the steps in conducting an overview. Multiple iterations of the framework were discussed and refined by all authors. In stage II, we identified studies evaluating methods and mapped these evaluations to the framework.

**Results:**

Forty-two stage I studies described methods relevant to one or more of the latter steps of an overview. Six studies evaluating methods were included in stage II. These mapped to steps involving (i) the assessment of risk of bias (RoB) in SRs (two SRs and three primary studies, all reporting evaluation of RoB tools) and (ii) the synthesis, presentation and summary of the findings (one primary study evaluating methods for measuring overlap).

**Conclusion:**

Many methods have been described for use in the latter steps in conducting an overview; however, evaluation and guidance for applying these methods is sparse. The exception is RoB assessment, for which a multitude of tools exist—several with sufficient evaluation and guidance to recommend their use. Evaluation of other methods is required to provide a comprehensive evidence map.

**Electronic supplementary material:**

The online version of this article (10.1186/s13643-018-0784-8) contains supplementary material, which is available to authorized users.

## Background

Overviews of systematic reviews aim to systematically retrieve, critically appraise and synthesise the results of multiple systematic reviews (SRs) [1]. Overviews of reviews (also called umbrella reviews, meta-reviews, reviews of reviews; but referred to in this paper as ‘overviews’ [2]) have grown in number in recent years, largely in response to the increasing number of SRs [3]. Overviews have many purposes including mapping the available evidence and identifying gaps in the literature, summarising the effects of the same intervention for different conditions or populations or examining reasons for discordance of findings and conclusions across SRs [4–6]. A noted potential benefit of overviews is that they can address a broader research question than the constituent SRs, since overviews are able to capitalise on previous SR efforts [7].

The steps and many of the methods used in the conduct of SRs are directly transferrable to overviews. However, overviews involve unique methodological challenges that primarily stem from a lack of alignment between the PICO (Population, Intervention, Comparison, Outcome) elements of the overview question and those of the included SRs, and overlap, where the same primary studies contribute data to multiple SRs [7]. For example, overlap can lead to challenging scenarios such as how to deal with discordant risk of bias assessments of the same primary studies across SRs (often further complicated by the use of different risk of bias/quality tools) or how to synthesize results from multiple meta-analyses where the same studies contribute to more than one pooled analysis. Authors need to plan for these scenarios, which may require the application of different or additional methods to those used in systematic reviews of primary studies.

Two recent reviews of methods guidance for conducing overviews found that there were important gaps in the guidance on the conduct of overviews [8, 9]. The results of our first paper—which identified methods for the initial steps in conducting an overview and collated the evidence on the performance of these methods [10]—aligned with these findings. We further identified that there was a lack of studies evaluating the performance of overview methods and limited empirical evidence to inform methods decision-making in overviews [10].

This paper is the second of two papers, which together, aim to provide a comprehensive framework of overview methods and the evidence underpinning these methods—an evidence map of overview methods. In doing so, we aim to help overview authors plan for common scenarios encountered when conducting an overview and enable prioritisation of methods development and evaluation.

## Objectives

The objectives of this study were to (a) develop a comprehensive framework of methods that have been used, or may be used, in conducting, interpreting and reporting overviews of systematic reviews of interventions (stage I)—the *M*ethods for *O*verviews *o*f *R*eviews (MOoR) framework; (b) map studies that have evaluated these methods to the framework (creating an evidence map of overview methods) (stage II); and (c) identify unique methodological challenges of overviews and methods proposed to address these.

**Fig. 1:**
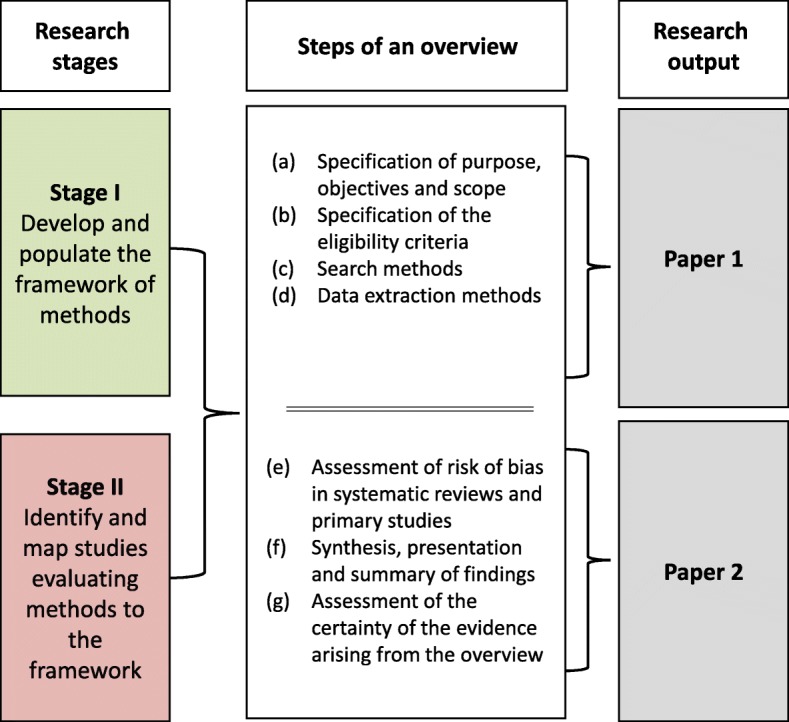
Summary of the research reported in each paper

In the first paper, we presented the methods framework, along with the studies that had evaluated those methods mapped to the framework (the evidence map) for the four initial steps of conducting an overview: (a) specification of the purpose, objectives and scope of the overview; (b) specification of the eligibility criteria; (c) search methods and (d) data extraction methods [10]. In this second companion paper, we present the methods framework and evidence map for the subsequent steps in conducting an overview: (e) assessment of risk of bias in SRs and primary studies; (f) synthesis, presentation and summary of the findings and (g) assessment of the certainty of evidence arising from the overview (Fig. 1).

We use the term ‘methods framework’ (or equivalently, ‘framework of methods’) to describe the organising structure we have developed to group-related methods, and against which methods evaluations can be mapped. The highest level of this structure is the broad steps of conducting an overview (e.g. synthesis, presentation and summary of the findings). The methods framework, together with the studies that have evaluated these methods, form the evidence map of overview methods.

## Methods

**Fig. 2:**
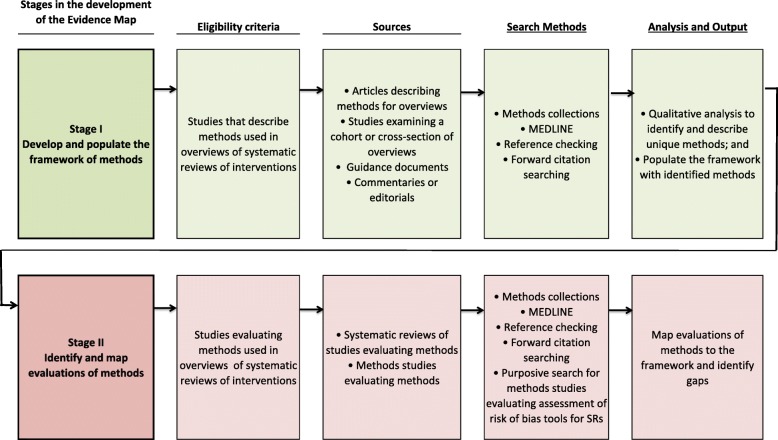
Stages in the development of an evidence map of overview methods

A protocol for this study has been published [11], and the methods have been described in detail in the first paper in the series [10]. The methods for the two research stages (Fig. 2) are now briefly described, along with deviations from the planned methods pertaining to this second paper. A notable deviation from our protocol is that we had planned to include the step ‘interpretation of findings and drawing conclusions’, but after reviewing the literature, felt that there was overlap between this step and the ‘assessment of certainty of the evidence arising from the overview’ step, and so consolidated the identified methods into the latter step.

### Stage I: development and population of the framework of methods

#### Search methods

Our main search strategy included searching MEDLINE from 2000 onwards and the following methods collections: Cochrane Methodology Register, Meth4ReSyn library, Scientific Resource Center Methods library of the AHRQ Effective Health Care Program and Cochrane Colloquium abstracts. Searches were run on December 2, 2015 (see Additional file 1 for search strategies). These searches were supplemented by methods articles we had identified through a related research project, examination of reference lists of included studies, contact with authors of conference posters, and citation searches (see Paper 1 [10] for details).

#### Eligibility criteria

We identified articles describing methods used, or recommended for use, in overviews of systematic reviews of interventions.

Inclusion criteria:

(i) Articles describing methods for overviews of systematic reviews of interventions
(ii) Articles examining methods used in a cross-section or cohort of overviews
(iii) Guidance (e.g. handbooks and guidelines) for undertaking overviews
(iv) Commentaries or editorials that discuss methods for overviews

Exclusion criteria:

(i) Articles published in languages other than English
(ii) Articles describing methods for network meta-analysis
(iii) Articles exclusively about methods for overviews of other review types (i.e. not of interventions)

We populated the framework with methods that are different or additional to those required to conduct a SR of primary research. Methods evaluated in the context of other ‘overview’ products, such as guidelines, which are of relevance to overviews, were included.

The eligibility criteria were piloted by three reviewers independently on a sample of articles retrieved from the search to ensure consistent application.

#### Study selection

Two reviewers independently reviewed the title, abstracts and full text for their potential inclusion against the eligibility criteria. Any disagreement was resolved by discussion with a third reviewer. In instances where there was limited or incomplete information regarding a study’s eligibility (e.g. when only an abstract was available), the study authors were contacted to request the full text or further details.

#### Data extraction, coding and analysis

One author collected data from all included articles using a pre-tested form; a second author collected data from a 50% sample of the articles.

##### Data collected on the characteristics of included studies

We collected data about the following: (i) the type of articles (coded as per our inclusion criteria), (ii) the main contribution(s) of the article (e.g. critique of methods), (iii) a precis of the methods or approaches described and (iv) the data on which the article was based (e.g. audit of methods used in a sample of overviews, author’s experience).

##### Coding and analysis to develop the framework of methods

We coded the extent to which each article described methods or approaches pertaining to each step of an overview (i.e. mentioned without description, described—insufficient detail to implement, described—implementable). The subset of articles coded as providing description were read by two authors (CL, SB or JM) who independently drafted the framework for that step to capture and categorise all available methods. We grouped conceptually similar approaches together and extracted examples to illustrate the options. Groups were labelled to delineate the unique decision points faced when planning each step of an overview (e.g. determine how to deal with discordance across systematic review (SR)/meta-analyses (MAs) and determine criteria for selecting SR/MAs, where SR/MAs include overlapping studies). To ensure comprehensiveness of the framework, methods were inferred when a clear alternative existed to a reported method (e.g. using tabular or graphical approaches to present discordance (6.2, Table 4)). The drafts and multiple iterations of the framework for each step were discussed and refined by all authors.

### Stage II: identification and mapping of evaluations of methods

#### Search methods

In addition to the main searches outlined in the ‘Search methods’ section for Stage I, we planned to undertake purposive searches to locate ‘studies evaluating methods’ where the main searches were unlikely to have located these evaluations. For this second paper, we undertook a purposive search to locate studies evaluating assessment of risk of bias tools for SRs, since these studies may not have mentioned ‘overviews’ (or its synonyms) in their titles or abstracts and thus would not have been identified in the main searches. However, through our main search, we identified a SR that had examined quality assessment or critical appraisal tools for assessing SRs or meta-analyses [12]. We therefore did not develop a new purposive search strategy, but instead used the strategy in the SR, and ran it over the period January 2013—August 2016 to locate studies published subsequent to the SR (Additional file 2). For the other steps, the identified methods were specific to overviews, so evaluations were judged likely to be retrieved by our main searches.

#### Eligibility criteria

To create the evidence map, we identified studies evaluating methods for overviews of systematic reviews of interventions.

Inclusion criteria:

(i) SRs of methods studies that have evaluated methods for overviews
(ii) Primary methods studies that have evaluated methods for overviews

Exclusion criteria:

(i) Studies published in languages other than English
(ii) Methods studies that have evaluated methods for network meta-analysis

We added the additional criterion that methods studies had to have a stated aim to evaluate methods, since our focus was on evaluation and not just application of a method.

#### Study selection

We used the same process, as outlined in the ‘Study selection’ section, for determining which studies located from the main search met the inclusion criteria. For studies located from the purposive search, one author reviewed title, abstracts and full text for their potential inclusion against the eligibility criteria.

#### Data extraction

We extracted data from primary methods studies, or SRs of methods studies that evaluated the measurement properties of tools for assessing the risk of bias in SRs and one study that developed measures to quantify overlap of primary studies in overviews. The data extracted from these studies were based on relevant domains of the COSMIN checklist (Table 1) [13, 14]. We had originally planned to extract quantitative results from the methods evaluations relating to the primary objectives; however, on reflection, we opted not to do this since we felt this lay outside the purpose of the evidence map. Data were extracted independently by three authors (CL, SM, SB, JM).

**Table 1:** Data extracted from methods studies evaluating tools for assessing risk of bias in SRs

Study design Category	Data extracted
**Primary methods studies**	
Study characteristics	First author, year
	Title
	Primary objective
Description of primary methods studies	Name of the included tools or measures
	Type of assessment (e.g. assessment of reliability, content validity)
	Content validity—methods of item generation
	Content validity—comprehensiveness
	Reliability—description of reliability testing
	Tests of validity description of correlation coefficient testing
	Other assessment (feasibility, acceptability, piloting)
Risk of bias criteria	Existence of a protocol
	Method to select the sample of SRs to which the tool/measure was applied
	Process for selecting the raters/assessors who applied the tool/measure
	Pre-specified hypotheses for testing of validity
**Systematic reviews of methods studies**	
Study characteristics	First author, year
	Title
Description of SRs of methods studies	Primary objective
	Number of included tools
	Number of studies reporting on the included tools
	Name of the included tools or measures (unnamed tools are identified by first author name and year of publication)
	Content validity—reported method of development (e.g. item generation, expert assessment of content)
	Reliability—description of reliability testing
	Construct validity—description of any hypothesis testing. For example, how assessments from two or more tools relate, whether assessments relate to other factors (e.g. effect estimates or findings)
	Other assessment (feasibility, acceptability, piloting)
Risk of bias criteria (using three domains from the ROBIS tool [15])	Domain 1—study eligibility criteria: concerns regarding specification of eligibility criteria (low, high or unclear concern)
	Domain 2—identification and selection of studies: concerns regarding methods used to identify and/or select studies (low, high or unclear concern)
	Domain 3—data collection and study appraisal: concerns regarding methods used to collect data and appraise studies (low, high or unclear concern)
	Overall judgment: Interpretation addresses all concerns identified in Domains 1–3, relevance of studies was appropriately considered, reviewers avoided emphasising results based on statistical significance.

#### Assessment of the risk of bias

For primary methods studies, we extracted and tabulated study characteristics that may plausibly be associated with either bias or the generalisability of findings (external validity) (Table 1). For SRs of methods studies, we used the ROBIS tool to identify concerns with the review process in the specification of study eligibility (Domain 1), methods used to identify and/or select studies (Domain 2), and the methods used to collect data and appraise studies (Domain 3) (Table 1) [15]. We then made an overall judgement about the risk of bias arising from these concerns (low, high, or unclear). We did not assess Domain 4 of ROBIS, since this domain covers synthesis methods that are of limited applicability to the included reviews.

#### Analysis

The yield, characteristics and description of the studies evaluating methods were described and mapped to the framework of methods.

## Results

### Results of the main search

**Fig. 3:**
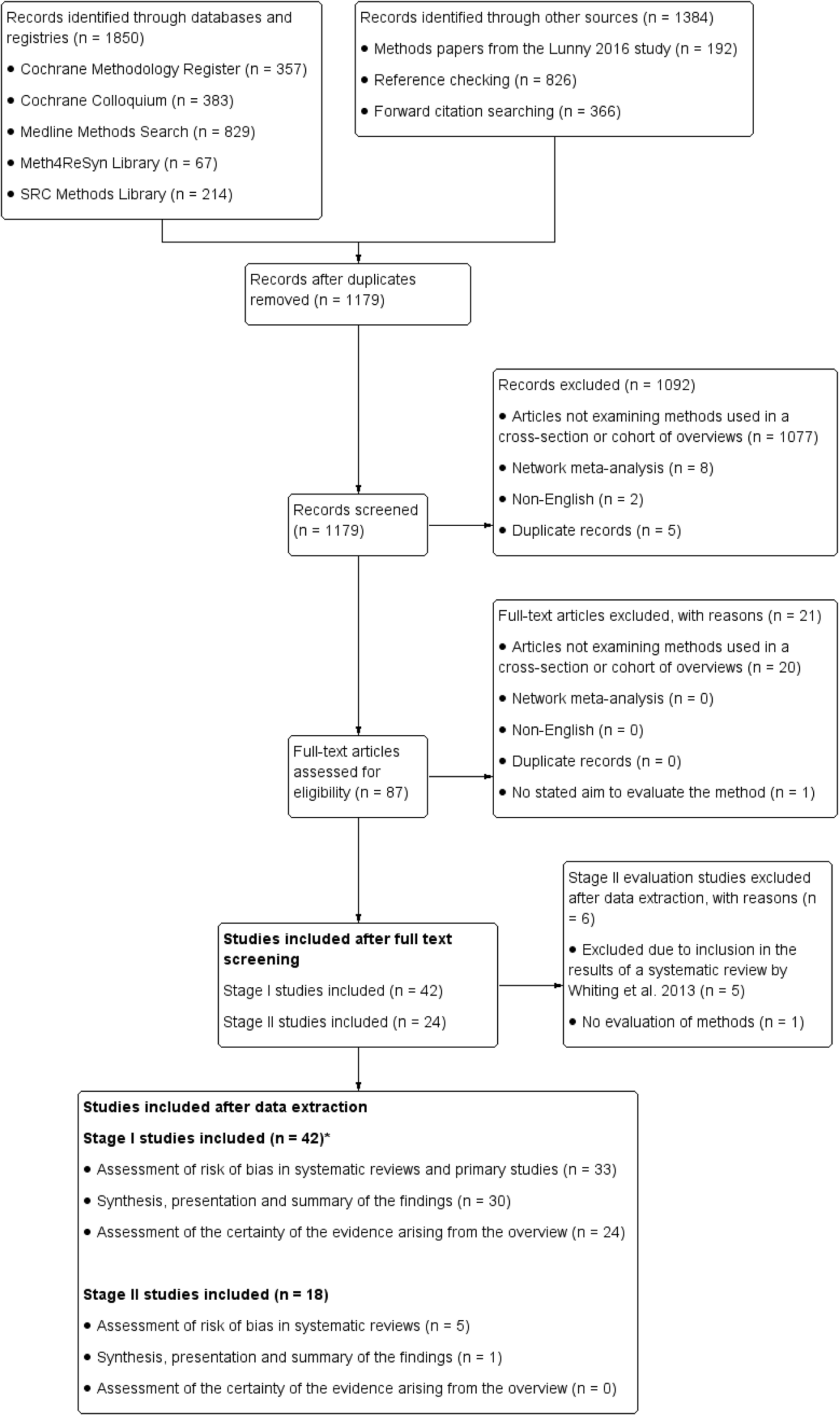
Flowchart of the main search for stages I and II studies

Details of our search results are reported in our first companion paper [10]. Here, we note the results from the additional purposive search and changes in search results between the papers. Our main search strategy retrieved 1179 unique records through searching databases, methods collections and other sources (Fig. 3) [10]. After screening abstracts and full text, 66 studies remained, 42 of which were included in stage I and 24 studies in stage II (exclusions found in Additional file 3). Our purposive search to identify studies evaluating tools for assessing the risk of bias in SRs (rather than primary studies) found no further stage II studies (see Additional file 4 for flowchart).

Of the 24 included stage II studies, 12 evaluated search filters for SRs (reported in paper 1 [10]), 11 evaluated risk of bias assessment tools for SRs, and one evaluated a synthesis method. Of the 11 studies evaluating risk of bias assessment tools for SRs, four were SRs of methods studies ([12, 16–18] and seven were primary evaluation studies [15, 17, 19–23].

Four of the seven primary evaluations of risk of bias assessment tools [20–23] and one SR [16] were included in the results of the 2013 SR by Whiting [12] and so were not considered individually in this paper. We excluded one of the SRs since, after close examination, it became clear that it reviewed studies that applied rather than evaluated AMSTAR (A Measurement Tool to Assess Systematic Reviews [22, 23]) and so did not meet our stage II inclusion criteria [18]. Therefore, of the 24 initially eligible stage II studies, 18 met the inclusion criteria, six of which are included in this second paper (Fig. 3).

### Stage I: development and population of the framework of methods

We first describe the characteristics of the included stage I articles (see ‘Characteristics of stage I articles’; Table 2) followed by presentation of the developed framework. This presentation is organised into sections representing the main (latter) steps in conducting an overview—‘assessment of risk of bias in SRs and primary studies’, ‘synthesis, presentation and summary of findings’ and the ‘assessment of certainty of the evidence arising from the overview’. In each section, we orient readers to the structure of the methods framework, which includes a set of steps and sub-steps (which are numbered in the text and tables). Reporting considerations for all steps are reported in Additional file 5.

**Table 2:** Characteristics of stage I studies and the extent to which each described (two ticks) or mentioned (one tick) methods pertaining to the latter steps in conducting an overview

Citation	Type of study	Summary description of the article	Latter steps in the conduct of an overview		
			Assessment of RoB in SRs and primary studies	Synthesis, presentation and summary of the findings	Assessment of the certainty of the evidence arising from the overview
Baker [43] The benefits and challenges of conducting an overview of systematic reviews in public health: a focus on physical activity.	Article describing methods for overviews	• Describes the usefulness of overviews for decision-makers and summarises some procedural steps to be undertaken • Provides a case study of an overview on public health interventions for increasing physical activity	✓	✓	
Becker [4] Overviews of reviews.	Guidance for undertaking overviews	• Early guidance providing the structure and procedural steps for the conduct of an overview • Details the RoB/quality assessment of SRs, and describes how to present findings through tables and figures	✓✓	✓	✓✓
Bolland [5] A case study of discordant overlapping meta-analyses: vitamin D supplements and fracture.	Article describing methods for overviews	• Describes criteria for explaining differences in SR/MAs addressing a similar question with discordant conclusions • Builds on the guide to interpret discordant SRs proposed by Jadad 1997 [29] • Suggests reporting items when an overview contains SR/MAs addressing a similar question	✓	✓	✓✓
Brunton [44] Putting the issues on the table: summarising outcomes from reviews of reviews to inform health policy.	Study examining methods used in a cohort of overviews	• Presents a tabular method of vote counting (positive effect/negative effect/no change) for each reported outcome		✓	
Büchter [45, 65] How do authors of Cochrane Overviews deal with conflicts of interest relating to their own systematic reviews?	Study examining methods used in a cohort of overviews	• Describes potential conflicts arising from dual authorship (where an overview author includes one or more SRs they authored) and suggests ‘safeguards’ to protect against potential bias • Describes reporting items in relation to dual authorship	✓		
Caird [1] Mediating policy-relevant evidence at speed: are systematic reviews of systematic reviews a useful approach?	Article describing methods for overviews	• Describes the methodological challenges in the production of overviews aimed at translating the knowledge to policy makers • Describes the trade-offs between producing a rapid overview and its comprehensiveness and reliability	✓✓	✓✓	✓✓
Chen [46] Scientific hypotheses can be tested by comparing the effects of one treatment over many diseases in a systematic review.	Study examining methods used in a cohort of overviews	• Identifies possible aims of an overview as being to detect unintended effects, improve the precision of effect estimates or explore heterogeneity of effect across disease groups • Describes the value and pitfalls of synthesis of MAs using three case studies	✓✓	✓✓	
CMIMG [47] Review type and methodological considerations.	Guidance for undertaking overviews	• Builds on the Cochrane guidance for overviews by Becker 2008 [4] • Describes the factors in the decision to conduct an overview vs. an SR • Describes how to handle potential overlap in primary studies across SRs and RoB/quality of SRs	✓	✓	
Cooper [6] The overview of reviews: unique challenges and opportunities when research syntheses are the principal elements of new integrative scholarship.	Article describing methods for overviews	• Describes steps in the conduct of an overview and methods to address challenges (e.g. dealing with overlap in primary studies across SRs) • Describes methods for second order meta-analysis	✓✓	✓✓	✓✓
Crick [48] An evaluation of harvest plots to display results of meta-analyses in overviews of reviews: a cross-sectional study	Article describing methods for overviews	• Examines the feasibility of using harvest plots as compared to summary tables to depict results of MAs in overviews		✓✓	✓
Flodgren [49] Challenges facing reviewers preparing overviews of reviews.^a^	Article describing methods for overviews	• Mentions the issue of missing or inadequately reported data • Mentions the challenges in summarising and evaluating large amounts of heterogeneous data across SRs	✓✓	✓	
Foisy [50] Mixing with the ‘unclean’: Including non-Cochrane reviews alongside Cochrane reviews in overviews of reviews.^a^	Article describing methods for overviews	• Mentions some challenges inherent in defining AMSTAR scoring as inclusion criteria, and inclusion of non-Cochrane reviews alongside Cochrane reviews • Mentions inclusion criteria to minimise overlap in primary studies across SRs	✓		
Foisy [51] Grading the quality of evidence in existing systematic reviews: challenges and considerations.	Study examining methods used in a cohort of overviews	• Mentions problems overview authors may encounter when applying GRADE to included SRs without going back to original data, and potential solutions			✓
Foisy [52] Challenges and considerations involved in using AMSTAR in overviews of reviews.	Article describing methods for overviews	• Describes differences in AMSTAR scores across Cochrane and non-Cochrane SRs, challenges in using AMSTAR to assess RoB of included SRs, and using the AMSTAR score as inclusion criterion	✓✓		
Hartling [53] A descriptive analysis of overviews of reviews published between 2000 and 2011.	Study examining methods used in a cohort of overviews	• Describes methodological standards for SRs (PRISMA, MECIR) and their applicability to overviews • Describes methods related to RoB of primary studies extracted from the SRs, RoB of the SRs, quantitative analysis and certainty of the evidence	✓✓	✓	✓✓
Hartling [54] Generating empirical evidence to support methods for overviews of reviews.^a^	Study examining methods used in a cohort of overviews	• Identifies methodological issues when conducting overviews • Mentions RoB of SRs, limitations of existing tools and challenges in assessing the certainty of the evidence	✓		✓
Hartling [55] Systematic reviews, overviews of reviews and comparative effectiveness reviews: a discussion of approaches to knowledge synthesis.	Article describing methods for overviews	• Briefly defines overviews, mentions the purposes in conducting an overview, and discusses some methodological challenges		✓	
Hemming [56] Pooling systematic reviews of systematic reviews: a Bayesian panoramic meta-analysis.	Article describing methods for overviews	• Proposes a Bayesian method for meta-analysis (hierarchical meta-analysis) which uses uninformative priors as a means of pooling effect estimates in an overview		✓✓	
Ioannidis [57] Integration of evidence from multiple meta-analyses: a primer on umbrella reviews, treatment networks and multiple treatments meta-analyses.	Article describing methods for overviews	• Defines ‘umbrella reviews’ as a pre-step to network meta-analysis • Describes challenges of overviews • Describes some reporting items for overviews		✓	
Jadad [29] A guide to interpreting discordant systematic reviews.	Article describing methods for overviews	• Seminal paper summarising the potential sources of discordance in results in a cohort of MAs, and types of discordance • Presents an algorithm for interpreting discordant SR results	✓	✓✓	✓✓
James [58] Informing the methods for public health overview reviews: a descriptive analysis of Cochrane and non-Cochrane public health overviews ^a^	Study examining methods used in a cohort of overviews	• Briefly describes several steps in the conduct of overviews • Compares Cochrane and non-Cochrane reviews in terms of tool used for assessing methodological quality, and certainty of the evidence	✓		✓
Joanna Briggs Institute (JBI) [39, 59] Methodology for JBI umbrella reviews.	Guidance for undertaking overviews	• Provides guidance as to what methods should be used at which step in the conduct of an overview • Provides stylistic conventions for overviews to meet publication and reporting criteria for the JBI Database of Systematic Reviews and Implementation Reports	✓✓	✓✓	✓
Kovacs [60] Overviews should meet the methodological standards of systematic reviews.	Commentary that discusses methods for overviews	• Mentions methodological challenges of overviews	✓	✓	
Kramer [61] Preparing an overview of reviews: lessons learned. ^a^	Article describing methods for overviews	• Mentions the challenges encountered when the authors conducted three overviews including dealing with heterogeneity	✓	✓	
Li [62] Quality and transparency of overviews of systematic reviews.	Article describing methods for overviews	• Presents a pilot reporting/quality checklist • Evaluates a cohort of overviews using the pilot tool, with the mean number of items but no details of the items	✓		
Moja [63] Multiple systematic reviews: methods for assessing discordances of results.	Article describing methods for overviews	• Describes methods to assess discordant finding among MAs based on the Jadad 1997 tool [29]		✓	✓✓
O’Mara [64] Guidelines for conducting and reporting reviews of reviews: dealing with topic relevances and double-counting.^a^	Article describing methods for overviews	• Presents a ‘utility’ rating based on the SRs PICO compared to the overviews’ PICO question • Mentions graphical and tabular approaches to establish the extent of overlap in primary studies across SRs		✓	
Pieper [3, 65] Overviews of reviews often have limited rigor: a systematic review.	Study examining methods used in a cohort of overviews	• Describes the methods used in overviews • Recommends using validated search filters for retrieval of SRs • Discusses whether to update the overview by including primary studies published after the most recent SR	✓	✓	✓
Pieper [66] Methodological approaches in conducting overviews: current state in HTA agencies.	Article describing methods for overviews	• Describes the methods recommended in 8 HTA guideline documents related to overviews • Compares the Cochrane Handbook guidance [67] to guidance produced by HTA agencies	✓✓	✓	✓
Pieper [68] Up-to-dateness of reviews is often neglected in overviews: a systematic review.	Study examining methods used in a cohort of overviews	• Describes the process of searching for additional primary studies in an overview • Presents decision rules for when to search for additional primary studies • Describes sequential searching versus parallel searching for retrieving SRs and primary studies in an overview	✓		✓✓
Pollock [31] An algorithm was developed to assign GRADE levels of evidence to comparisons within systematic reviews.	Article describing methods for overviews	• Adapts GRADE guidance to assess the certainty of the evidence for a specific overview • Presents an algorithm to assess certainty of the evidence	✓		✓✓
Robinson [24, 69–72] Integrating bodies of evidence: existing systematic reviews and primary studies.	Article describing methods for overviews	• Describes the steps and methods to undertake a complex review that includes multiple SRs, which is similar to methods used in overviews • Describes methods for assessing risk of bias of primary studies in SRs, and methods to assess the certainty of the evidence	✓✓	✓✓	✓✓
Ryan [25] Building blocks for meta-synthesis: data integration tables for summarising, mapping, and synthesising evidence on interventions for communicating with health consumers.	Article describing methods for overviews	• Presents tabular methods to deal with the preparation of overview evidence • Discusses the data extraction process and organisation of data • Presents a table of taxonomy of outcomes from the included SRs and a data extraction table based on this taxonomy	✓✓	✓✓	✓✓
Salanti [73] Evolution of Cochrane intervention reviews and overviews of reviews to better accommodate comparisons among multiple interventions.	Guidance for undertaking overviews	• Defines overviews as integrating or synthesising (rather than summarising) evidence from SRs • Describes methods for synthesising multiple-intervention SRs		✓	
Schmidt [74] Methods for second order meta-analysis and illustrative applications.	Article describing methods for overviews	• Describes statistical methods for second-order meta-analysis • Provides examples of modelling between-MA variation		✓✓	
Silva [75] Overview of systematic reviews—a new type of study. Part II	Study examining methods used in a cohort of overviews	• Examines a cohort of Cochrane reviews for methods used • Documented the sources and types of search strategies conducted	✓		✓✓
Singh [76] Development of the Metareview Assessment of Reporting Quality (MARQ) Checklist.	Article describing methods for overviews	• Presents a pilot reporting/quality checklist • Evaluates four case studies using the pilot tool, with the mean number of items but no details of the items	✓✓	✓	
Smith [77] Methodology in conducting a systematic review of systematic reviews of healthcare interventions.	Article describing methods for overviews	• Describes some steps and challenges in undertaking an overview, namely eligibility criteria, search methods, and RoB/quality assessment • Presents tabular methods for presentation of results in an overview	✓✓	✓✓	✓✓
Tang [78] A statistical method for synthesizing meta-analyses.	Article describing methods for overviews	• Describes statistical methods for second-order MA with examples		✓✓	
Thomson [26] The evolution of a new publication type: steps and challenges of producing overviews of reviews.	Article describing methods for overviews	• Describes some steps in undertaking an overview and some of the challenges in conducting an overview • Discusses that gaps or lack of currency in included evidence will weaken the overview findings	✓✓	✓✓	✓✓
Thomson [79] Overview of reviews in child health: evidence synthesis and the knowledge base for a specific population.	Study examining methods used in a cohort of overviews	• Describes the process of including trials in overviews • Discusses the challenge of overview PICO differing from the PICO of the included SRs • Provides potential solutions as to what to do when mixed populations are reported in SRs and how to extract subgroup data		✓	
Wagner [80] Assessing a systematic review of systematic reviews: developing a criteria.	Article describing methods for overviews	• Presents a quality assessment tool for appraisal of overviews	✓		✓

*AHRQ’s EPC* Agency for Healthcare Research and Quality’s Evidence-based Practice Center; *AMSTAR* A Measurement Tool to Assess Systematic Reviews; *CMIMG* Comparing Multiple Interventions Methods Group; *GRADE* Grading of Recommendations Assessment, Development, and Evaluation; *HTA* health technology assessment; *JBI* Joanna Briggs Institute; *MA* meta-analysis; *MECIR* Methodological Expectations of Cochrane Intervention Reviews; *PICO* Population, Intervention, Comparison, Outcome; *PRISMA* Preferred Reporting Items for Systematic Reviews and Meta-Analyses; *RoB* risk of bias; *R-AMSTAR* revised AMSTAR; *SR* systematic review

^a^Indicates a poster presentation

✓✓ Indicates a study describing one or more methods

✓ Indicates a study mentioning one or more methods

We focus our description on methods/options that are distinct; have added complexity, compared with SRs of primary studies; or have been proposed to deal with major challenges in undertaking an overview. Importantly, the methods/approaches and options reflect the ideas presented in the literature and should not be interpreted as endorsement for the use of the methods. We also highlight methods that may be considered for dealing with commonly encountered scenarios for which overview authors need to plan (see ‘Addressing common scenarios unique to overviews’; Table 6).

#### Characteristics of stage I articles

The characteristics and the extent to which articles (*n* = 42) described methods pertaining to the latter steps in conducting an overview are indicated in Table 2. The majority of articles were published as full reports (*n* = 34/42; 81%). The most common type of study was an article describing methods for overviews (*n* = 26/42; 62%), followed by studies examining methods used in a cohort of overviews (*n* = 11/42; 26%), guidance documents (*n* = 4/42; 10%) and commentaries and editorials (*n* = 1/42; 2%).

Methods for the assessment of risk of bias in SRs and primary studies were most commonly mentioned or described (*n* = 33), followed by methods for synthesis, presentation and summary of the findings (*n* = 30), and methods for the assessment of certainty of the evidence in overviews (*n* = 24). Few articles described methods across all of the latter steps in conducting an overview (*n* = 6 [1, 4, 6, 24–26]).

#### Assessment of risk of bias in SRs and primary studies

The three steps in the framework under ‘assessment of risk of bias in SRs and primary studies’ were ‘plan to assess risk of bias (RoB) in the included SRs (1.0)’, ‘plan how the RoB of the primary studies will be assessed or re-assessed (2.0)’ and ‘plan the process for assessing RoB (3.0)’ (Table 3). Note that in the following we use the terminology ‘risk of bias’, rather than quality, since assessment of SR or primary study limitations should focus on the potential of those methods to bias findings. However, the terms quality assessment and critical appraisal are common, particularly when referring to the assessment of SR methods, and hence, our analysis includes all relevant literature irrespective of terminology. We now highlight methods/approaches and options for the first two steps since these involve decisions unique to overviews.

**Table 3:** Assessment of risk of bias in SRs and primary studies

Step Sub-step Methods/approaches	Sources (first author, year) ▪ Examples
1.0 Plan to assess risk of bias (RoB) in the included SRs^§^	
1.1 Determine how to assess RoB in the included SRs	
1.1.1 Select an existing RoB assessment tool for SRs	Baker 2014 [43]; Becker 2008 [4]; Bolland 2014 [5]; Büchter 2011 [45, 65]; Caird 2015 [1]; Chen 2014 [46]; CMIMG 2012 [47]; Cooper 2012 [6]; Flodgren 2011 [49]; Foisy 2011 [50]; Foisy 2014 [52]; Hartling 2012 [53]; Hartling 2013 [54]; Jadad 1997 [29]; James 2014 [58]; JBI 2014 [39, 59]; Kovacs 2014 [60]; Kramer 2009 [61]; Li 2012 [62]; Pieper 2012 [3]; Pieper 2014c [66]; Pieper 2014d [68]; Pieper 2014a [17]; Robinson 2015 [24, 69–72]; Ryan 2009 [25]; Silva 2014 [75]; Singh 2012 [76]; Smith 2011 [77]; Thomson 2010 [26]; Whiting 2013 [12]
1.1.2 Adapt an existing RoB tool (e.g. selecting or modifying items for the overview)	CMIMG 2012 [47]; Hartling 2012 [53]; Jadad 1997 [29]; Pollock 2015 [31] ▪ Pollock 2015 assessed 4 (of 11) AMSTAR items thought to be the most important sources of bias, and developed sub-questions for each [31] ▪ Reporting selected items/domains modifies the tool, since some items/domains are ignored [53]
1.1.3 Develop a RoB tool customised to the overview	CMIMG 2012 [47]; Cooper 2012 [6]; Pieper 2012 [3]; Pieper 2014a [17]
1.1.4 Use existing RoB assessments	Baker 2014 [43] ▪ Use quality assessments of SRs published by Health Evidence^TM^ [27] or Health Systems Evidence [81]
1.1.5 Describe characteristics of included SRs that may be associated with bias or quality without using or developing a tool	Pieper 2014a [17]; Robinson 2015 [24, 69–72]
1.2 Determine how to summarise or score the RoB assessments for SRs	
1.2.1 Report assessment for individual items or domains (with or without rationale for judgements)	Hartling 2012 [53]
1.2.2 Summarise assessments across items or domains by using a scoring system^§§^	JBI 2014 [39, 59]; Pieper 2014a [17]; Robinson 2015 [24, 69–72]; Whiting 2013 [12]; Ryan 2009 [25]; Silva 2014 [75] ▪ Sum items, assigning equal or unequal weight to each (JBI 2014 [39, 59]) ▪ Calculate the mean score across items (JBI 2014 [39, 59])
1.2.3 Summarise assessments across items or domains, then use cut-off scores or thresholds to categorise RoB using qualitative descriptors (e.g. low, moderate or high quality)^§§^	Crick 2013 [48]; JBI 2014 [39, 59]; Robinson 2015 [24, 69–72]; Ryan 2009 [25]; Silva 2014 [75]; Singh 2012 [76] ▪ Pollock 2015 [31] set cut-offs for rating an SR as having no serious limitations (‘yes’ response to 4/4 AMSTAR items), serious limitations (‘yes’ to 3/4 items and 1 ‘unclear’), or very serious limitations (‘yes’ to < 3/4) ▪ SRs that score < 3/10 on the AMSTAR scale might be considered low quality, 4-6/10 moderate quality, and 7–10/10 high quality (JBI 2014 [39, 59]) ▪ All domains/items required (all domains/items required for SR to be deemed low RoB)
1.3 Determine how to present the RoB assessments for SRs	
1.3.1 Display assessments in table(s) (e.g. overall rating in summary of findings table, and another table with RoB items for each SR)	Aromataris 2015 [39, 59]; Becker [4]; Chen 2014 [46]; Hartling 2012 [53]; Smith 2011 [77]
1.3.2 Display assessments graphically	Crick 2015 [48] ▪ ROBIS RoB graph depicting authors’ judgments about each domain presented as percentages across all included SRs [15] ▪ Harvest plot, which depicts results according to study size and quality ([48])
1.3.3 Report assessments in text	Aromataris 2015 [39, 59]; Chen 2014 [46]; Hartling 2012 [53]; Li 2012 [62]
2.0 Plan how the RoB of primary studies will be assessed or re-assessed	
2.1 Determine how to assess the RoB of the primary studies in the included SRs (and any additional primary studies)	
2.1.1 Report RoB assessment of primary studies from the included SRs, using the approaches specified for data extraction to deal with missing, flawed assessments, or discrepant/discordant assessments of the same primary study (i.e. where two or more SRs assess the same study using different tools or report discordant judgements using the same tool) (See ‘Data extraction’ table in [10]).	Aromataris 2015 [39, 59]; Becker 2008 [4]; Caird 2015 [1]; CMIMG 2012 [47]; Cooper 2012 [6]; Hartling 2012 [53]; Hartling 2014 [55]; Jadad 1997 [29]; Ioannidis 2009 [57]; Kramer 2009 [61]; Ryan 2009 [25]; Singh 2012 [76]; Thomson 2010 [26] ▪ Report RoB assessments of primary studies from the included SR(s), noting missing data and discrepancies (Hartling 2012 [53]; JBI 2014 [39]; Robinson 2015 [24, 69–72] ▪ Report RoB assessments from the highest quality SR (Jadad 1997 [29])
2.1.2 Report RoB assessment of primary studies from the included SRs after performing quality checks to verify that the assessment method has been applied appropriately and consistently across a sample of primary studies	Becker 2008 [4]; Hartling 2014 [55]; Ioannidis 2009 [57]; Jadad 1997 [29]; Kramer 2009 [61]; Moja 2012 [63]; Robinson 2015 [24, 69–72]; Thomson 2010 [26] ▪ Randomly sample a number of included RCTs, retrieve data from the original trial reports, and independently check 10% of RCT data from the included MAs to verify assessments were done without error and consistently ▪ Repeat RoB assessments on a sample of SRs to verify and check for consistency (Robinson 2015 [24, 69–72])
2.1.3 (Re)-assess RoB of some or all primary studies^a^	CMIMG 2012 [47]; Cooper 2012 [6]; Hartling 2012 [53]; Jadad 1997 [29]; Moja 2012 [63]; Thomson 2010 [26] ▪ When two different tools are used, then assess the primary studies using one tool ▪ When two different tools are used (e.g. Cochrane RoB tool [67] and Jadad tool [29]; then re-assess RoB by standardising the assessments based on the Cochrane RoB domains, and match data from assessments from other tools to these domains)
2.1.4 Don’t report or assess RoB of primary studies	Inferred
2.2 Determine how to summarise the RoB assessments for primary studies	
2.2.1 Report assessment for individual items or domains (with or without rationale for judgements)^a^	JBI 2014 [39, 59]; Pieper 2014c [66]; Robinson 2015 [24, 69–72]; Ryan 2009 [25]; Silva 2014 [75]
2.2.2 Summarise assessments across items or domains by using a scoring system^§§^	JBI 2014 [39, 59]; Pieper 2014c [66]; Robinson 2015 [24, 69–72]; Ryan 2009 [25]; Silva 2014 [75] ▪ Sum items, assigning equal or unequal weight to each (JBI 2014 [39, 59]) ▪ Calculate the mean score across items (JBI 2014 [39, 59])
2.2.3 Summarise assessments across items or domains, then use cut-off scores or thresholds to describe RoB (e.g. low, moderate and high quality)^§§^	JBI 2014 [39, 59]; Robinson 2015 [24, 69–72]; Ryan 2009 [25]; Silva 2014 [75]; Singh 2012 [76]
2.3 Determine how to present the RoB assessments for primary studies	
2.3.1 Display assessments in table(s) (e.g. overall rating in summary of findings table, and another table with RoB items for each primary study)^a^	Aromataris 2015 [39, 59]; Becker 2008 [4]; Chen 2014 [46]; Hartling 2012 [53]; Smith 2011 [77]; JBI 2014 [39, 59]
2.3.2 Display assessments graphically^a^	Crick 2015 [48] ▪ Cochrane RoB graph depicting authors’ judgments about each domain presented as percentages across all included SRs [67] ▪ Harvest plot, which depicts results according to study size and quality ([48])
2.3.3 Report assessments in text^a^	Aromataris 2015 [39, 59]; Chen 2014 [46]; Hartling 2012 [53]; Li 2012 [62]; Smith 2011 [77]
3.0 Plan the process for assessing RoB	
3.1 Determine the number of overview authors required to assess studies^a^	
3.1.1 Independent assessment by 2 or more authors	Baker 2014 [43]; Becker 2008 [4]; Cooper 2012 [6]; JBI 2014 [39]; Li 2012 [62]; Ryan 2009 [25]
3.1.2 One author assessment	Inferred
3.1.3 One assessment, 2nd confirmed	Cooper 2012 [6]
3.1.4 One assessment, 2nd confirms if uncertainty	Cooper 2012 [6]
3.2 Determine if authors (co-)authored one or several of the SRs included in the overview, and if yes, plan safeguards to avoid bias in RoB assessment	Büchter 2011 [45, 65] ▪ Assessment of RoB of included SRs done by overview authors who were not authors of the SRs

*AMSTAR* A Measurement Tool to Assess Systematic Reviews; *CMIMG* Comparing Multiple Interventions Methods Group; *JBI* Joanna Briggs Institute; *OQAQ* Overview Quality Assessment Questionnaire; *RoB* risk of bias; *ROBIS* Risk of Bias In Systematic reviews; *SRs* systematic reviews

^§^We refer to ‘risk of bias’ assessment, since assessment of SR or primary study limitations should focus on the potential of those methods to bias findings. However, the terms quality assessment and critical appraisal are common, particularly when referring to the assessment of SR methods, and hence our analysis includes all relevant literature irrespective of terminology

^§§^As is the case with assessment of RoB in primary studies, concerns have been raised about the validity of presenting a summary score or qualitative descriptors based on scores (e.g. low, moderate, high quality) [12, 17]

^a^Adaptation of the step from SRs to overviews. No methods evaluation required, but special consideration needs to be given to unique issues that arise in conducting overviews

When determining how to assess the RoB in SRs (1.1), identified approaches included the following: selecting or adapting an existing RoB assessment tool for SRs (1.1.1, 1.1.2), developing a RoB tool customised to the overview (1.1.3), using an existing RoB assessment such as those published in Health Evidence^TM^ [27] (1.1.4) or describing the characteristics of included SRs that may be associated with bias or quality without using or developing a tool (1.1.5). More than 40 tools have been identified for appraisal of SRs [12], only one of which is described as a risk of bias tool (ROBIS (Risk of Bias In Systematic reviews tool) [15]). Other tools are described as being for critical appraisal or quality assessment. Studies have identified AMSTAR [22, 23] and the OQAQ (Overview Quality Assessment Questionnaire [28]) as the most commonly used tools in overviews [3, 12]. Methods for summarising and presenting RoB assessments mirror those used in a SR of primary studies (1.2, 1.3).

Authors must also decide on how to assess the RoB of primary studies included within SRs (2.0). Two main approaches were identified: to either report the RoB assessments from the included SRs (2.1.1) or to independently assess RoB of the primary studies (2.1.3) (only the latter option applies when additional primary studies are retrieved to update or fill gaps in the coverage of existing SRs). When using the first approach, overview authors may also perform quality checks to verify assessments were done without error and consistently (2.1.2). In attempting to report RoB assessments from included SRs, overview authors may encounter missing data (e.g. incomplete reporting of assessments) or assessments that are flawed (e.g. using problematic tools). In addition, discrepancies in RoB assessments may be found when two or more SRs report an assessment of the same primary study but use different RoB tools or report discordant judgements for items or domains using the same tool. We identified multiple methods for dealing with these scenarios, most are applied at the data extraction stage (covered in Paper 1 [10]). Options varied according to the specific scenario, but included the following: (a) extracting all assessments, recording discrepancies; (b) extracting from one SR based on a priori criteria; (c) extracting data elements from the SR that meets pre-specified decision rules and (d) retrieving primary studies to extract missing data or reconcile discrepancies ([10]).

#### Synthesis, presentation and summary of the findings

The six steps in the framework under ‘synthesis, presentation and summary of the findings’ were ‘plan the approach to summarising the SR results (1.0)’, ‘plan the approach to quantitatively synthesising the SR results (2.0)’ ‘plan to assess heterogeneity (3.0)’, ‘plan the assessment of reporting biases (4.0)’, ‘plan how to deal with overlap of primary studies included in more than one SR (5.0)’, and ‘plan how to deal with discordant results, interpretations and conclusions of SRs (6.0)’ (Table 4). As a note on terminology, we distinguish between discrepant data—meaning data from the same primary study that differs between what is reported in SRs due to error in data extraction, and discordant results, interpretation and conclusions of the results of SRs—meaning differences in results and conclusions of SRs based on the methodological decisions authors make, or different interpretations or judgments about the results.

**Table 4:** Synthesis, presentation and summary of the findings

Step Sub-step Methods/approaches	Sources (first author, year) ▪ Examples
1.0 Plan the approach to summarising the SR results	
1.1 Determine criteria for selecting SR results/MAs, where SR/MAs include overlapping studies	
1.1.1 Include all SR results/MAs	Caird 2015 [1]; Cooper 2012 [6]
1.1.2 Use decision rules or tools (e.g. Jadad tool [29]) to select results from a subset of SR/MAs	Caird 2015 [1]; Cooper 2012 [6] ▪ Select one SR result/MA from overlapping SR/MAs based on (a) the MA with the most complete information, and if that was equivalent, (b) the MA with the largest number of primary studies (Cooper 2012 [6])
1.2 Determine the summary approach	
1.2.1 Describe and/or tabulate the characteristics of the included SRs in terms of PICO elements	Becker 2008 [4]; Cooper 2012 [6]; JBI 2014 [39, 59]; Pieper 2014c [66]; Robinson 2015 [24, 69–72]; Ryan 2009 [25]; Smith 2011 [77]; Thomson 2010 [26] ▪ Matrix of studies by PICO elements to allow comparison and assess important sources of heterogeneity across the SRs (Caird 2015 [1]; Kramer 2009 [61]; Smith 2011 [77]; Thomson 2010 [26])
1.2.2 Describe and/or tabulate the results of the included SRs	Becker 2008 [4]; Caird 2015 [1]; Chen 2014 [46]; Cooper 2012 [6]; Hartling 2012 [53]; JBI 2014 [39, 59]; Pieper 2014c [66]; Robinson 2015 [24, 69–72]; Ryan 2009 [25]; Salanti 2012 [73]; Silva 2014 [75]; Singh 2012 [76]; Smith 2011 [77]; Thomson 2010 [26] ▪ Present pooled effect estimates and their confidence intervals (and associated statistics such as estimates of heterogeneity, *I*^2^), number and types of studies, number of participants, meta-analysis model and estimation method, authors conclusions ▪ Present the forest plots from the included SRs (Chen 2014 [46]; Pieper 2014c [66])
1.2.3 Describe and/or tabulate the results of the included primary studies, including new or additional primary studies ^a^	Caird 2015 [1]; Cooper 2012 [6]; O’Mara 2011 [64]; Robinson 2015 [24, 69–72] ▪ For example, summary data, effect estimates and their confidence intervals, study design, number of study participants (O’Mara 2011 [64])
1.2.4 Summarise and/or tabulate RoB assessments of SRs and primary studies	Becker 2008 [4]; Caird 2015 [1]; Chen 2014 [46]; Hartling 2012 [53]; JBI 2014 [39, 59]; Li 2012 [62]; Ryan 2009 [25]; Robinson 2015 [24, 69–72]; Smith 2011 [77] ▪ For example, summarise the RoB/quality assessment methods used across the SRs
1.2.5 Summarise and/or tabulate results from any investigations of statistical heterogeneity (e.g. results from subgroup analyses / meta-regression) within the included SRs	Cooper 2012 [6]; JBI 2014 [39, 59]; Smith 2011 [77]
1.2.6 Summarise and/or tabulate results from any investigations of reporting biases (e.g. results from statistical tests for funnel plot asymmetry) within the included SRs	Singh 2012 [76]; Smith 2011 [77] ▪ Tabulate statistical tests of publication bias from the included MAs (Smith 2011 [77])
1.2.7 Determine the order of reporting the results in text and tables (e.g. by outcome domain, by effectiveness of interventions)^a^	Becker 2008 [4]; Bolland 2014 [5]; Salanti 2011 [73]; Smith 2011 [77]
1.2.8 Determine methods for converting or standardising effect metrics (either from primary studies or meta-analyses) to the same scale (e.g. odds ratios to risk ratios)^a^	Becker 2008 [4]; Cooper 2012 [6]; Thomson 2010 [26] ▪ Where a variety of summary statistics, such as odds ratios and risk ratios, are reported across SR/MAs, convert the results into one summary statistic to facilitate interpretation and comparability among results (Thomson 2010 [26])
1.2.9 Determine methods to group results of specific outcomes (from either primary studies or MAs) into broader outcome domains^a^	Ryan 2009 [25]; Thomson 2010 [26] ▪ Use an existing outcome taxonomy (e.g. Cochrane Consumers and Communication Review Group’s taxonomy). For example, results of an intervention on specific outcomes knowledge, accuracy, and risk of perception all map to the outcome domain consumer knowledge and understanding (Ryan 2009 [25])
1.3 Determine graphical approaches to present the results^a^	Becker 2008 [4]; Chen 2014 [46]; Crick 2015 [48]; Hartling 2014 [55]; JBI 2014 [39, 59]; Pieper 2014c [66]; Pieper 2014a [17] ▪ Use a forest plot to present MA effects (95% CI) from each SR sometimes referred to as ‘forest top plot’ (Becker 2008 [4]; Pieper 2014a [17]) ▪ Use a harvest plot to present the direction of effect for trials or MAs or both, also depicting study size and quality (Crick 2015 [48]) ▪ Use a bubble plot to display three dimensions of information, using colour to differentiate clinical indications: the *x*-axis (e.g. meta-analytic effect size), *y*-axis (e.g. SR quality), and the size of the bubble (e.g. number of included primary studies in a SR) ▪ Use a network plot to present the treatments that have been compared, with nodes representing treatments and links between nodes representing comparisons between treatments (Cooper 2012 [6])
2.0 Plan the approach to quantitatively synthesising the SR results	
2.1 Do not conduct a new quantitative synthesis (e.g. because of lack of time or resources)	Salanti 2011 [73]
2.2 Specify triggers for when to conduct a new quantitative synthesis	
2.2.1 Need to combine results from multiple MAs (with non-overlapping studies) for the same comparison and outcome	Robinson 2015 [24, 69–72]
2.2.2 Need to incorporate additional primary studies; or, incorporate these studies under certain circumstances	Robinson 2015 [24, 69–72]; Pieper 2014a [17] ▪ When the identified SRs are out of date and more recent primary studies have been published (Robinson 2015 [24, 69–72]) ▪ When inclusion of primary studies may change conclusions, strength of evidence judgements, or add new information (e.g. a trial undertaken in a population not currently included in the overview)
2.2.3 Need to apply new meta-analysis methods, fitting a more appropriate meta-analysis method and model, or using a different effect metric	Robinson 2015 [24, 69–72] ▪ When a new meta-analysis method such as prediction intervals are required ▪ When a fixed effect model was fitted in a SR, but a random effects model was more appropriate ▪ When a risk ratio is used instead of an odds ratio
2.2.4 Need to limit or expand the MAs into a new MA that meets the population, intervention and comparator elements of the overview	Thomson 2010 [26]; Whitlock 2008 [24, 69–72] ▪ Extracting the subset of trials that include only children and adolescents from a MA that includes trials with no restriction on age
2.2.5 Need to undertake a new meta-analysis because of concerns regarding the trustworthiness of the SR/MA results	Robinson 2015 [24, 69–72] ▪ Concerns regarding data extraction errors
2.2.6 Need to conduct a MA (if possible and makes sense to do so) because the SRs did not undertake MA	Inferred
2.2.7 Need to conduct a MA to reconcile discordant findings of previous SRs	White 2009 [24, 69–72] ▪ If overview authors cannot determine reasons for the discordant findings among SRs, then they can regard this as an indication that they need to conduct a new MA (White 2009 [24, 69–72])
2.3 Determine the meta-analysis approach	
2.3.1 Undertake a first-order meta-analysis of effect estimates (meta-analysis of the primary study effect estimates)^a^	Becker 2008 [4]; Chen 2014 [46]; Cooper 2012 [6]; Pieper 2014a [17]; Robinson 2015 [24, 69–72]; Schmidt 2013 [74]; Tang 2013 [78]; Thomson 2010 [26] ▪ May re-extract data from the primary studies, or use the data reported in the reviews (see ‘Data extraction’ table in [10])
2.3.2 Undertake a second-order meta-analysis of effect estimates (meta-analysis of meta-analyses) either ignoring the potential correlation across the meta-analysis estimates (arising from the same study included in more than one meta-analysis), or applying an adjustment to account for the potential correlation (e.g. inflating the variance of the meta-analysis)	Caird 2015 [1]; Chen 2014 [46]; Cooper 2012 [6]; Hemming 2012 [56]; Schmidt 2013 [74]; Tang 2013 [78] ▪ This issue of potential correlation (or non-independence) of the meta-analysis effect estimates may be more of a concern in overviews that seek to undertake a meta-analysis of the effects for the *same* intervention and *same* population, as compared with undertaking a meta-analysis of effects *across* populations (with the latter sometimes referred to as panoramic or multiple-indication reviews) (Chen 2014 [46]; Hemming 2012 [56]) ▪ Refer to 5.1.4 for statistical approaches to dealing with overlap
2.3.3 Undertake vote counting (e.g. based on direction of effect)^a^	Becker 2008 [4]; Caird 2015 [1]; Flodgren 2011 [49]; Ryan 2009 [25]; Tang 2013 [78]; Thomson 2010 [26]
2.4 Determine the method to convert effect metrics (either from primary studies or meta-analyses) to the same scale^a^	Cooper 2012 [6]; Tang 2013 [78]; Thomson 2010 [26]
2.5 Determine the meta-analysis model and estimation methods^a^	Cooper 2012 [6]; Hemming 2012 [56]; Schmidt 2013 [74] ▪ For example, second order meta-analysis: fixed or random effects model to combine meta-analytic effects (Schmidt 2013 [74]) ▪ For example, first-order meta-analysis across clinical conditions (multiple indication, panoramic review): three level hierarchical model, mixed effects model (Chen 2014 [46]; Hemming 2012 [56]) ▪ For example, parametric or non-parametric methods (Cooper 2012 [6]) ▪ For example, DerSimonian and Laird between-study variance estimator (Robinson 2015 [24, 69–72]; Tang 2013 [78])
2.6 Determine graphical approaches^a^	Becker 2008 [4]; Chen 2014 [46]; Crick 2015 [48]; Li 2012 [62]; Pieper 2014a [17]; Pieper 2014c [66] ▪ Use forest plots—either of meta-analysis results from each review, or results from individual studies (Becker 2008 [4]; Pieper 2014a [17]; Chen 2014 [46]; Pieper 2014c [66]; ▪ Use a harvest plot, which depicts results according to study size and quality, noting the direction of effect (Crick 2015 [48])
3.0 Plan to assess heterogeneity	
3.1 Determine summary approaches	
3.1.1 Tabulate results by modifying factors (e.g. study size, quality)^a^	Caird 2015 [1]; Chen 2014 [46]; Hartling 2012 [53]; JBI 2014 [39, 59]; Singh 2012 [76] ▪ Graph or tabulate results of SRs by modifying factors (e.g. group by the type of included study design [SRs of RCTs, SRs of observational studies); group by methodological quality of the SRs, their completeness in evidence coverage, or how up-to-date they are) (Caird 2015 [1]; Chen 2014 [46]; Hartling 2012 [53]; JBI 2014 [39, 59])
3.1.2 Graph results by modifying factors^a^	(Caird 2015 [1]; Chen 2014 [46]; Hartling 2012 [53]; JBI 2014 [39, 59])
3.2 Determine approach to identifying and quantifying heterogeneity^a^	Cooper 2012 [6] ▪ Visual examination of overlap of confidence intervals in the forest plot, *I*^2^ statistic, chi-squared test for heterogeneity
3.3 Determine approach to investigation of modifiers of effect in meta-analyses	
3.3.1 Undertake a first-order subgroup analysis of primary study effect estimates^a^	Becker 2008 [4]; Chen 2014 [46]; Cooper 2012 [6]; Singh 2012 [76]; Robinson 2015 [24, 69–72]; Thomson 2010 [26]
3.3.2 Undertake a second-order subgroup analysis of meta-analysis effect estimates with moderators categorised at the level of the meta-analysis (e.g. SR quality). Issues of correlation across the meta-analysis estimates may occur (see 2.3.2)	Cooper 2012 [6]
3.4 Determine the meta-analysis model and estimation methods^a^	Refer to 2.5 ▪ For example, random effects meta-regression
4.0 Plan the assessment of reporting biases	
4.1 Determine non-statistical approaches to assess missing SRs	Pieper 2014d [68]; Singh 2012 [76] ▪ Search SR registers (e.g. PROSPERO) ▪ Search for SR protocols
4.2 Determine non-statistical approaches to assess missing primary studies	Bolland 2014 [5] ▪ Identify non-overlapping primary studies across SRs and examine reasons for non-overlap (e.g. different SR inclusion / exclusion criteria, different search dates, different databases) as a method for discovering potentially missing primary studies from SRs (Bolland 2014 [5]) ▪ Conduct searches of trial registries to identify missing studies
4.3 Determine statistical methods for detecting and examining potential reporting biases from missing primary studies or results within studies, or selectively reported results^a^	Caird 2015 [1]; JBI 2014 [39, 59]; Singh 2012 [76]; Schmidt 2013 [74]; Smith 2011 [77] ▪ Visual assessment of funnel plot asymmetry of results from primary studies ▪ Statistical tests for funnel plot asymmetry using results from primary studies
5.0 Plan how to deal with overlap of primary studies included in more than one SR	
5.1 Determine methods for quantifying overlap	Cooper 2012 [6]; Pieper 2014b [35] ▪ Statistical measures to quantify the degree of overlap of primary studies across SRs (Pieper 2014b [35])
5.2 Determine how to visually examine and present overlap of the primary studies across SRs	Caird 2015 [1]; Chen 2014 [46]; Cooper 2012 [6]; JBI 2014 [39, 59]; O’Mara 2011 [64]; Robinson 2015 [24, 69–72]; Thomson 2010 [26] ▪ Display a matrix comparing which primary studies were included in which SRs; or other visual approaches demonstrating overlap (e.g. Venn diagrams as referenced in Patnode [82])
5.3 Determine methods for dealing with overlap	
5.3.1 Use decision rules, or a tool, to select one (or a subset of) MAs with overlapping studies (see also 1.1.2 above)	Caird 2015 [1]; Chen 2014 [46]; Cooper 2012 [6]; O’Mara 2011 [64]; Pieper 2012 [3]; Robinson 2015 [24, 69–72]; Thomson 2010 [26] ▪ Choose the meta-analyses with the most complete information; methodologically rigorous; recentness of the meta-analysis; inclusion of certain study types (e.g. only randomised trials); publication status ▪ Exclude SRs that do not contain any unique primary studies, when there are multiple SRs (Pieper 2014a [17]) ▪ Use a published algorithm or tool [Jadad 1997 [29]]
5.3.2 Use statistical approaches to deal with overlap	Cooper 2012 [6]; Tang 2013 [78] ▪ Identify meta-analyses with 25% or more of their research in common and eliminate the one with the fewer studies in each comparison, except when multiple smaller meta-analyses (with little overlap) would include more studies if the largest meta-analysis was eliminated (Cooper 2012 [6]) ▪ Sensitivity analyses (e.g. second-order MA including all MAs irrespective of overlap compared with second-order MA including only MAs where there is no overlap in primary studies) (Cooper 2012 [6]) ▪ Inflate the variance of the meta-analysis estimate (Tang 2013 [78])
5.3.3 Ignore overlap among primary studies in the included SRs	Cooper 2012 [6]; Caird 2015 [1]
5.3.4 Acknowledge overlap as a limitation	Caird 2015 [1]
6.0 Plan how to deal with discordant results, interpretations and conclusions of SRs	
6.1 Determine methods for dealing with or reporting discordance across SRs	
6.1.1 Examine and record discordance among SRs addressing a similar question	Caird 2015 [1]; Chen 2014 [46]; Cooper 2012 [6]; Hartling 2012 [53]; JBI 2014 [39, 59]; Kramer 2009 [61]; Pieper 2014c [66]; Pieper 2012 [3]; Robinson 2015 [24, 69–72]; Smith 2011 [77]; Thomson 2010 [26] ▪ Discordance among SRs can arise from a lack of overlap in studies, or methodological differences
6.1.2 Use decision rules or tools (e.g. Jadad 1997 [29]) to select one (or a subset of) SR/MAs	Bolland 2014 [5]; Caird 2015 [1]; Chen 2014 [46]; Cooper 2012 [6]; Hartling 2012 [53]; Jadad 1997 [29]; JBI 2014 [39, 59]; Kramer 2009 [61]; Moja 2012 [63]; Pieper 2012 [3]; Pieper 2014c [66]; Robinson 2015 [24, 69–72]; Smith 2011 [77]; Tang 2013 [78]; Thomson 2010 [26] ▪ Use a published algorithm based on whether the reviews address the same question, are of the same quality, have the same selection criteria (Jadad 1997 [29]) ▪ Use an adapted algorithm (pre-existing algorithm adapted for the overview) (Bolland 2014 [5])
6.2 Determine tabular or graphical approaches to present discordance	Inferred

*JBI* Joanna Briggs Institute; *MA* meta-analyses; *PICOs* Population (*P*), intervention (*I*), comparison (*C*), outcome (*O*), and study design (*s*); *PROSPERO* International Prospective Register of Systematic Reviews; *RCT* randomised controlled trial; *SRs* systematic reviews

^a^Adaptation of the step from SRs to overviews. No methods evaluation required, but special consideration needs to be given to unique issues that arise in conducting overviews

An identified step of relevance to all overviews is determining the summary approach (1.2). This includes determining what data will be extracted and summarised from SRs and primary studies (e.g. characteristics of the included SRs (1.2.1), results of the included SRs (1.2.2), results of the included primary studies (1.2.3), RoB assessments of SRs and primary studies (1.2.4)) and what graphical approaches might be used to present the results (1.3). In overviews that include multiple SRs reporting results for the same population, comparison and outcome, criteria need to be determined as to whether all SR results/MAs are reported (1.1.1), or only a subset (1.1.2). When the former approach is chosen (1.1.1), methods for dealing with overlap of primary studies across SR results need to be considered (5.0), such as acknowledging (5.3.4), statistically quantifying (5.1) and visually examining and depicting the overlap (5.2). Choice of a subset of SR/MAs (1.1.2) may bring about simplicity in terms of summarising the SR results (since there will only be one or a few SRs included), but may lead to a loss of potentially important information through the exclusion of studies that are not overlapping with the selected SR result(s).

A related issue is that of discordance (6.0). Some overviews aim to compare results, conclusions and interpretations across a set of SRs that address similar questions. These overviews typically address a focused clinical question (e.g. comparing only two interventions for a specific condition and population). Identified methods included approaches to examine and record discordance (6.1.1) and the use of tools (e.g. Jadad [29]) or decision rules to aid in the selection of one SR/MA (6.1.2).

In addition to determining the summary approach of SR results, consideration may also be given to undertaking a new quantitative synthesis of SR results (2.0). A range of triggers that may lead to a new quantitative synthesis were identified (2.2) (e.g. incorporation of additional primary studies (2.2.2), need to use new or more appropriate meta-analysis methods (2.2.3), concerns regarding the trustworthiness of the SR/MA results (2.2.5)). When undertaking a new meta-analysis in an overview, a decision that is unique to overviews is whether to undertake a first-order meta-analysis of effect estimates from primary studies (2.3.1), or a second-order meta-analysis of meta-analysis effect estimates from the SRs (2.3.2). If undertaking a second-order meta-analysis, methods may be required for dealing with primary studies contributing data to multiple meta-analyses (5.3.2). A second-order subgroup analysis was identified as a potential method for investigating whether characteristics at the level of the meta-analysis (e.g. SR quality) modify the magnitude of intervention effect (3.3.2). If new meta-analyses are undertaken, decisions regarding the model and estimation method are required (2.5, 3.4).

Investigation of reporting biases may be done through summarising the reported investigations of reporting biases in the constituent SRs (1.2.6), or through new investigations (4.0). Overviews also provide an opportunity to identify missing primary studies through non-statistical approaches (4.2), such as comparing the included studies across SRs. An additional consideration in overviews is investigation of missing SRs. Identified non-statistical approaches to identify missing SRs included searching SR registries and protocols (4.1).

#### Assessment of the certainty of the evidence arising from the overview

The two steps in the framework under ‘assessment of the certainty of the evidence arising from the overview’ are as follows: ‘plan to assess certainty of the evidence (1.0)’ and ‘plan the process for assessing the certainty of the evidence (2.0)’ (Table 5). GRADE is the most widely used method for assessing the certainty of evidence in a systematic review of primary studies. The methods involve assessing study limitations (RoB, imprecision, inconsistency, indirectness, and publication bias) to provide an overall rating of the certainty of (or confidence in) results for each comparison [30]. In an overview, planning how to assess certainty (1.1) involves additional considerations. These include deciding how to account for limitations of the included SRs (e.g. bias arising from the SR process, whether SRs directly address the overview question) and how to deal with missing or discordant data needed to assess certainty (e.g. non-reporting of heterogeneity statistics needed to assess consistency, SRs that report conflicting RoB assessments for the same study). One approach is to assess certainty of the evidence using a method designed for overviews (1.1.1). However, GRADE methods (or equivalent) have not yet been adapted for overviews and guidance on addressing issues is not available. In the absence of agreed guidance for overviews, another option is to assess the certainty of the evidence using an ad hoc method (1.1.2). For example, Pollock 2015 incorporated the limitations of included SRs in their GRADE assessment by rating down the certainty of evidence for SRs that did not meet criteria deemed to indicate important sources of bias [31, 32].

**Table 5:** Assessment of the certainty of the evidence arising from the overview

Step Sub-step Methods/approaches	Sources (first author, year) ▪ Examples
1.0 Plan to assess certainty of the evidence	
1.1 Determine how to assess the certainty of the evidence	
1.1.1 Assess the certainty of the evidence using a method developed for use in overviews	Wagner 2012 [80] ▪ Wagner 2012 [80] report an approach to assigning levels of evidence in an overview based on the number and quality of included SRs (primary studies were not considered).
1.1.2 Assess the certainty of the evidence using an ad hoc method developed for a specific overview	Bolland 2014 [5]; Cooper 2012 [6]; Crick 2015 [48]; Hartling 2012 [53]; Pollock 2015 [31]; Ryan 2009 [25]; Thomson 2010 [26]; Wagner 2012 [80] ▪ Pollock 2015 [31] adapted GRADE methods for their overview, incorporating an additional domain to account for potential bias arising from the methods used in included SRs. Decision rules were used to ensure consistent grading of domains deemed important to their overview question; these did not specifically address considerations unique to overviews
1.1.3 Report assessments of certainty of the evidence from the included SRs, using the approaches specified for data extraction to deal with missing data, flawed or discordant assessments (e.g. where two SRs use different methods to assess certainty of the evidence or report discordant assessments using the same method) (see ‘Data extraction’ table in [10]).	Becker 2008 [4]; Cooper 2012 [6]; Hartling 2012 [53]; Hartling 2014 [55]; JBI 2014 [39, 59]; Kramer 2009 [61]; Pieper 2014c [66]; Robinson 2015 [24, 69–72]; Ryan 2009 [25]; Silva 2014 [75] ▪ Report assessments of the certainty of the evidence for each comparison and outcome directly from the included SRs, irrespective of the method used, noting missing data and discrepancies (Hartling 2012 [53]; JBI 2014 [39]; Robinson 2015 [24, 69–72]) ▪ Report the certainty of the evidence data from the Cochrane review with the most comprehensive assessment
1.1.4 Report assessments of certainty of the evidence from the included SRs after performing quality checks on a sample of assessments to verify that the assessment method has been applied appropriately and consistently across SRs	Becker 2008 [4]; Robinson 2015 [24, 69–72]; Thomson 2010 [26] ▪ Report the certainty of the evidence assessments after retrieving primary study data from the included trials and independently check 10% of primary study data ▪ Report the certainty of the evidence assessments after cross-checking the assessments across overlapping SRs (Becker 2008 [4]; and quoted in Thomson 2010 [26])
1.1.5 (Re)-assess the certainty of the evidence using an existing method developed for SRs of primary studies without adapting the method for overviews	Crick 2015 [48]; Foisy 2014 [51]; JBI 2014 [39]; Hartling 2012 [53]; Robinson 2015 [24, 69–72]; Ryan 2009 [25]; Thomson 2010 [26] ▪ Use GRADE [30] for assessing the certainty of the evidence without modifying the domains or decision rules used to assess the certainty of the evidence in a SR of primary studies (Hartling 2012 [53]; JBI 2014 [39, 59]; Robinson 2015 [24, 69–72]). May be done for missing assessments, if there are missing studies from an assessment, if there are concerns about reported assessment(s), or if there are differences between the overview and SR questions that necessitate re-assessment (e.g. different population). ▪ For new primary studies or those not integrated into the assessment reported in SRs, re-assess the certainty of evidence (Hartling 2012 [53]; JBI 2014 [39, 59]; Robinson 2015 [24, 69–72]) ▪ When two different tools are used (e.g. GRADE [30] and AHRQ [33], then re-assess certainty of the evidence for each comparison and outcome by standardising the assessments based on similar domains
1.1.6 Do not report or assess the certainty of the evidence	Inferred
2.0 Plan the process for assessing certainty	
2.1 Determine the number of overview authors required to assess the certainty of the evidence^a^	
2.1.1 Independent assessment by 2 or more authors	Baker 2014 [43]; Becker 2008 [4]; Cooper 2012 [6]; Li 2012 [62]; JBI 2014 [39, 59]; Ryan 2009 [25]
2.1.2 One author assesses	Inferred
2.1.4 One assesses, 2nd confirms	Cooper 2012 [6]
2.1.5 One assesses, 2nd confirms if the first author is unsure	Cooper 2012 [6]
2.2 Determine if authors (co-)authored one or several of the SRs included in the overview, and if yes, plan safeguards to avoid bias in certainty of the evidence assessment	Büchter 2011 [45, 65] ▪ Overview authors do not assess the certainty of the evidence from their co-authored SRs

*AHRQ* Agency for Healthcare Research and Quality; *CMIMG* Comparing Multiple Interventions Methods Group; *GRADE* Grading of Recommendations Assessment, Development, and Evaluation; *JBI* Joanna Briggs Institute; *SRs* systematic reviews

^a^Adaptation of the step from SRs to overviews. No methods evaluation required, but special consideration needs to be given to unique issues that arise in conducting overviews

Other identified approaches use methods developed for SRs of primary studies, without adaptation for overviews. The simplest of these is to ‘report assessments of certainty of the evidence from the included SRs’ with or without checking accuracy first (1.1.3 and 1.1.4). Authors may then use approaches specified in the data extraction step to deal with missing or discrepant assessments (see paper 1 [10]). These approaches include simply noting missing data and discrepant assessments, or reporting assessments of certainty from an SR that meets pre-specified methodological eligibility criteria, for example, the review that addressed the overview question most directly or assessed to be at lowest risk of bias. The final option when using methods developed for SRs of primary studies involves completing the assessment of certainty from scratch (1.1.5). This option may apply in circumstances where (a) an assessment was not reported in included SRs, (b) new primary studies were retrieved that were not included in the SRs or relevant studies were not integrated into the assessment reported in the SR, (c) included SRs used different tools to assess certainty (e.g. GRADE [30] and the Agency for Healthcare Research and Quality’s [AHRQ] tool [33]) or (d) assessments are judged to be flawed or inappropriate for the overview question.

#### Addressing common scenarios unique to overviews

In our examination of the literature, methods were often proposed in the context of overcoming common methodological scenarios. Table 6 lists the methods options from the framework that could be used to address each scenario.

**Table 6:** Methods and approaches for addressing common scenarios unique to overviews

	Scenario for which authors need to plan	Methods/approaches proposed in the literature^a^		
		Assessment of RoB in SRs and primary studies (Table 3)	Synthesis, presentation and summary of the findings (Table 4)	Assessment of certainty of the evidence (Table 5)
1	Reviews include *overlapping* information and data (e.g. arising from inclusion of the same primary studies)	2.1.1	1.1.2, 5.0	1.1.1–1.1.5
2	Reviews report *discrepant* information and data	2.1.1, 2.1.2, 2.1.3	2.2.1, 2.2.5	1.1.1–1.1.5
3	Data are *missing* or reviews report *varying* information (e.g. information on risk of bias is missing or varies across primary studies because reviews use different tools)	2.1.1, 2.1.3	1.2.9, 2.2.1, 2.2.5	1.1.1–1.1.5
4	Reviews provide incomplete coverage of the overview question (e.g. missing comparisons, populations)		2.2.1, 2.2.4	1.1.1, 1.1.2, 1.1.5
5	Reviews are not up-to-date		2.2.2	1.1.1, 1.1.2
6	Review methods raise concerns about bias or quality	2.1.1, 2.1.2, 2.2.3	2.2.3, 2.2.5, 4.0	1.1.1–1.1.5
7	Reviews report *discordant* results and conclusions		2.2.7, 6.0	1.1.1–1.1.5

^a^The methods/approaches could be used in combination and at several steps in the conduct of an overview. When one approach is taken, then another approach may not apply

While the literature reviewed often suggested a single method or step at which a scenario should be dealt with, Table 6 shows that there are multiple options, some of which can be combined. Only those methods that provide direct solutions are listed, not those that need to be implemented as a consequence of the chosen solution. Taking an example, a commonly cited approach for dealing with reviews with overlapping primary studies is to specify eligibility criteria (or decision rules) to select one SR (see Paper 1 [10]). However, multiple methods exist for addressing overlap at later steps of the overview. During synthesis, for example, authors can (i) use decision rules to select one (or a subset) of meta-analyses with overlapping studies (5.3.1), (ii) use statistical approaches to deal with overlap (5.3.2), (iii) ignore overlap (5.3.3) or (iv) acknowledge overlap as a limitation (5.3.4; Table 4). Alternatively, overlap may be addressed when assessing certainty of the evidence. Any of these approaches can be combined with methods to quantify and visually present overlap (5.1–5.2; Table 4).

### Stage II: identification and mapping of evaluations of methods

#### Mapping studies evaluating methods to the framework

Five studies, published between 2011 and 2015, evaluated tools to assess risk of bias in SRs. Two were SRs [12, 17] and three were primary studies not included in either of the SRs [15, 19, 34]. Characteristics of these studies are summarised in Tables 7 and 8. All five studies map to the sub-option ‘select an existing RoB assessment tool for SRs’ (1.1.1) of the approach ‘plan to assess RoB in the included SRs’ (1.0) under the ‘assessment of RoB in SRs and primary studies’ step of the framework (see ‘Assessment of risk of bias in SRs and primary studies’; Table 3).

**Table 7:** Characteristics of SRs of methods studies and assessment of risk of bias

	Study ID (first author, year)	
	Pieper 2014a [17]	Whiting 2013 [12]
**Characteristics of the studies**		
Title	Systematic review found AMSTAR, but not R(evised)-AMSTAR, to have good measurement properties	Review of existing quality assessment tools for systematic reviews (Chapter 4)
Primary objective	To review all empirical studies evaluating the measurement properties of AMSTAR and R-AMSTAR	To conduct a review of existing tools designed to critically appraise SRs and meta-analyses. The review was conducted to inform development of ROBIS
Number of included tools	2	40 (5/40 tools targeted areas other than SRs of interventions, for example diagnostic test accuracy or genetic association studies)
Number of studies reporting on the included tools	13 (10 reporting on AMSTAR, 2 on R-AMSTAR, 1 on both) • 4/13 studies had a primary objective to assess the properties of AMSTAR/R-AMSTAR • 9/13 were methods studies that applied AMSTAR/R-AMSTAR (mainly assessing quality of SRs in a clinical area)	43
Name of the included tools or measures (unnamed tools are identified by first author name and year of publication)	AMSTAR [22, 23], R-AMSTAR [36]	Named tools: AMSTAR [22, 23], CASP [83], FOCUS [84], MAC [85], NHMRC [86], OQAQ [28], SIGN [87], RAPiD [88]^a^ Unnamed tools: Assendelft 1995 [89], Auperin 1997 [90], Crombie 1996 [91], Geller 1996 [92], Glenny 2003 [93], Greenhalgh 1997 [94], Higgins 2013 [38], Ho 2010 [95], Irwig 1994 [96], Knox 2009 [97], Li 2012 [98], Light 1984 [99], Lundh 2012 [100], Mailis 2012 [101], Minelli 2009 [102], Mokkink 2009 [14], Mulrow 1987 [103], Nony 1995 [104], Oxman 1988 [105], Oxman 1994 [106], Oxman 1994 [107] (3 tools), Philibert 2012 [108], Sacks 1997 [109], Santaguida 2012 [110], Shamliyan 2010 [111], Sheikh 2007 [112], Smith 1989 [12]; Smith 1997 [113], Smith 2007 [12], Thacker 1996 [114], Wilson 1992 [115], Zambon 2012 [116]
Content validity–reported method of development (e.g. item generation, expert assessment of content)	Not assessed (noted in background that AMSTAR was based on OQAQ and a checklist by Sacks 1997)	Methods of development were reported for 17/40 tools: • 3 tools were developed using a ‘rigorous’ process (AMSTAR, Higgins 2013, OQAQ)^§^ • 10 tools were based on multiple existing tools and/or guidelines for the conduct of systematic reviews (or similar) • 4 tools were adapted from a single tool ^§^OQAQ was based on literature review, survey of methodological experts, and pretesting (pilot study). AMSTAR was based on existing tools (including OQAQ), a consensus process aimed at establishing face and content validity, and exploratory factor analysis. Higgins 2013 was based on AMSTAR, the Cochrane Handbook for Systematic Reviews of Interventions [67], expert review of items, and pilot testing.
Reliability—description of reliability testing	Inter-rater reliability (IRR) assessments were reported in 11/13 studies, (9 on AMSTAR, 2 on R-AMSTAR). IRR results were reported for individual items (8 studies), the mean across all items (7 studies), and overall score (6 studies)	Inter-rater reliability assessments were reported for 5/40 tools (most reporting kappa or i*ntraclass correlation coefficient*)
Tests of validity—description of correlation coefficient testing	Six studies assessed construct validity examining the correlation between total AMSTAR scale scores (summing ‘yes’ responses) and scores on OQAQ (3 studies), Sack’s list (1 study), R-AMSTAR (1 study), and expert assessment (2 studies)	No tests of validity were reported for any tools (although exploratory factor analysis was used during development of content for AMSTAR)
Other assessments (feasibility, acceptability, piloting)	Time taken to complete tool	The SR includes a summary of tool content (items and domains measured), tool structure (e.g. checklist, domain based), and item rating (i.e. response options)
**Risk of bias in the SRs of methods studies**		
Domain 1—study eligibility criteria^b^	Low Unclear if predefined criteria/objectives were adhered to, but eligibility criteria are broad (lessening inappropriate exclusions), unambiguous and appropriate.	Low Unclear if predefined criteria/objectives were adhered to, but eligibility criteria are broad (lessening inappropriate exclusions), unambiguous and appropriate.
Domain 2—identification and selection of studies^b^	Low Comprehensive search of multiple databases and reference lists. While search terms are not reported in full, the authors searched for evaluations of specific tools, terms for which were likely to be reported in the abstract. Independent screening of citations and full text by two authors.	Low Comprehensive search. Independent screening of citations, single screening of full text with checks.
Domain 3—data collection and study appraisal^b^	High Single data extraction, with checks. COSMIN [13] was used to defined measurement properties and as a guide to interpreting findings, but not to appraise study methods. There is potential that the methods used for inter-rater reliability assessment may bias estimates of reliability; given this and the extent of reporting of reliability statistics, concern for this domain was rated as high.	Low Single data extraction, with checks. Most potential for error in extracting and classifying content of items, however the impact of misclassification is low. No assessments of risk of bias of included studies, but this is only a concern for studies that reported estimates of measurement properties (5/40 studies reported reliability statistics). Since interpretation of results focused on tool development and content, concern for this domain was rated as low.
Overall judgement^c^	Low risk of bias Although there is potential for bias in the reported estimates of reliability and validity, the authors were cautious in their interpretation, and noted the limitations of both the evaluations reported in included studies and their review methods.	Low risk of bias

*AMSTAR* A Measurement Tool to Assess Systematic Reviews; *CASP* Critical Appraisal Skills Programme; *IRR* Inter-rater reliability; *OQAQ* Overview Quality Assessment Questionnaire; *MAC* Meta-analysis Appraisal Checklist; *NHMRC* National Health and Medical Research Council; *RAPiD* Rapid Appraisal Protocol internet Database; *ROBIS* Risk of Bias In Systematic reviews; *SIGN* Scottish Intercollegiate Guidelines Network; *SRs* systematic reviews

^a^OQAQ [28] is also referred to as OQAC (Overview Quality Assessment Checklist), and RAPiD [88] is also referred to as RAP (Rapid Appraisal Protocol).

^b^Level of concern for each domain judged as low, high or unclear

^c^Overall judgement is based on: interpretation address all concerns identified in domains 1–3, relevance of studies was appropriately considered, reviewers avoided emphasising results based on statistical significance

**Table 8:** Characteristics of primary methods studies and assessment of risk of bias

	Study ID (first author, year)			
	Pieper 2014e [34]	Whiting 2016 [15]	Parmelli 2011 [19]	Pieper 2014b [35]
**Characteristics of the studies**				
Title	Impact of choice of quality appraisal tool for systematic reviews in overviews	ROBIS: A new tool to assess risk of bias in systematic reviews was developed	Using AMSTAR to assess the methodological quality of systematic reviews: An external validation study	Systematic review finds overlapping reviews were not mentioned in every other overview
Primary objective	To examine reliability, validity and feasibility of four quality appraisal tools in an SR and explore how the choice of tool impacts the findings of the evidence synthesis	To develop ROBIS, a new tool for assessing the risk of bias in systematic reviews (rather than in primary studies)	To measure the reliability, construct validity and feasibility of AMSTAR on a sample of SRs in different medical fields	To develop two measures to quantify the degree of overlap of primary studies across SRs and evaluate the validity of the measures
Name of the included tools or measures	AMSTAR [22, 23, 37], AQASR [117], OQAQ [28], RAPiD [88]^a^	ROBIS [15]	AMSTAR [22, 23], OQAQ	CA and CCA [35]
Type of assessment	Assess reliability/ construct validity of the tool	Assess content validity/reliability	Assess reliability/ construct validity of the tool	Construct validity testing of the measures
Content validity—methods of item generation	Not applicable—existing tool	Content (domains and items) was based on a reporting standard for SRs (i.e. MECIR [118]) and an SR of 40 tools designed to assess the quality of SRs or meta-analyses	Not applicable—existing tool	Not applicable—not a tool
Content validity—comprehensiveness	Not applicable—existing tool	Content experts (methodologists, systematic reviewers, guideline developers) reviewed the draft ROBIS tool in a face-to-face meeting and Delphi process	Not applicable—existing tool	Not applicable—not a tool
Reliability—description of reliability testing	Inter-rater reliability (agreement) between two review authors who independently applied AMSTAR, OQAQ, RAPiD and AQASR to 32 SRs. A 4-week interval separated assessment with each tool. Agreement was assessed at item level for AMSTAR and OQAQ, and domain level for RAP and AQASR (Cohen’s kappa)	Inter-rater reliability (agreement) between two review authors who independently applied ROBIS to 8 SRs. Agreement was assessed at domain level (% agreement)	Inter-rater reliability (agreement) between two review authors who independently applied AMSTAR to 54 SRs. Agreement was assessed at item level (Cohen’s weighted kappa)	Not applicable
Tests of validity—description of correlation coefficient testing	Correlation between summary scores on OQAQ and RAPiD (not done for tools without summary scores). Qualitative assessment of whether assessment of SR quality with different tools altered overall conclusions about strength of association between volume and outcomes (where SR quality was one of four elements used to determine strength)	Not assessed	Correlation between scores on AMSTAR and scores from a similar measure, the OQAQ (Pearson’s rank correlation coefficient, results not reported in abstract)	Correlation between measures (CA, CCA) calculated on a sample of overviews (Kendall τ-b) with each other, and each measure with the number of SRs and number of primary publications. Examined whether the measures were associated with publication source (HTA or journal publication), hypothesizing that HTA reports may have more overlap
Other assessment (feasibility, acceptability, piloting)	Time to complete	Piloting involved three workshops on ROBIS where participants piloted the tools and provided feedback	Time to complete	Not reported
**Risk of bias in the primary methods studies**				
Existence of a protocol	Not reported	Not reported	Not reported	Not reported
Method to select the sample of SRs to which the tool/measure was applied	Convenience: SRs were included studies in an overview that examined associations between surgery volume and outcomes (not intervention effects)	Convenience: SRs were included studies in an overview, being conducted by authors independent of the developers of ROBIS	Convenience: SRs were in two different medical fields (hypertension, colorectal cancer), and described as a convenience sample but unclear how they were selected	Census: All overviews identified from a literature search of five databases. Handsearching of websites of HTA agencies. Search restricted to articles published between 2009 and 2011.
Process for selecting the raters/assessors who applied the tool/measure^b^	Convenience: Raters were authors of an overview in which AMSTAR was used	Convenience: Raters were authors of an overview in which ROBIS was piloted, and were independent of the tool developers. Unclear how they were recruited	Unclear: No description of how raters were selected	Not applicable
Pre-specified hypotheses for testing of validity	No: The expected direction or magnitude of correlation was not specified. ‘The Spearman’s rank correlation coefficient was calculated to compare the CATs [critical appraisal tool]’	Not applicable: no testing of validity	No: The expected direction or magnitude of correlation was not specified. ‘Construct validity was investigated comparing the two instruments using Pearson’s rank correlation coefficient.’	Yes: ‘We hypothesized that the CA should have a strong (0.60–0.80) negative correlation with the number of included reviews and, compared to this, a lower negative correlation with the number of included primary publications. In contrast, we assumed that the CCA should have a very weak (0.00–0.20) or weak (0.20–0.40) negative correlation with the number of included reviews and the primary publications.’

*AMSTAR* A Measurement Tool to Assess Systematic Reviews; *AQASR* Assessing the Quality and Applicability of Systematic Reviews; *CA* Covered Area; *CCA* Corrected Covered Area; *HTA* Health Technology Assessment; *MECIR* Methodological Expectations of Cochrane Intervention Reviews; *OQAQ* Overview Quality Assessment Questionnaire; *RAPiD* Rapid Appraisal Protocol internet Database; *RoB* risk of bias; *ROBIS* Risk of Bias In Systematic reviews; *SRs* systematic reviews

^a^OQAQ [28] is also referred to as OQAC (Overview Quality Assessment Checklist), and RAPiD [88] is also referred to as RAP (Rapid Appraisal Protocol)

^b^RoB in relation to any estimates of reliability and validity

We found one study that evaluated methods for synthesis. Pieper 2014b developed and validated two measures to quantify the degree of overlap in primary studies across multiple SRs [35]. This study maps to the ‘synthesis, presentation and summary of the findings’ step of the framework (see ‘Synthesis, presentation and summary of the findings’; Table 4) in option 5.0 ‘plan how to deal with overlap of primary studies included in more than one SR’.

We found no stage II studies evaluating methods in the ‘assessment of the certainty of evidence arising from the overview’ step of the framework (Table 5).

Two SRs reviewed published tools to assess the risk of bias in SRs [12, 17]. Pieper [17] reviewed evidence of the reliability and construct validity of the AMSTAR [22, 23] and R-AMSTAR (revised-AMSTAR [36]) tools. Whiting [12] reviewed the content and measurement properties of 40 critical appraisal tools (Table 7). The review includes a summary of tool content (items and domains measured), tool structure (e.g. checklist, domain based), and item rating (i.e. response options). Studies included in Whiting [12] reported methods of development for 17 of 40 tools (i.e. providing information needed to assess content validity). Three of these 17 tools were judged to have been developed using a ‘rigorous’ process (notably AMSTAR [22, 23, 37], Higgins [38], and OQAQ [28]) (details in Table 7). Inter-rater reliability assessments were available from 11 of 13 studies included in Pieper [17], and for five of the 40 tools (most reporting kappa or intraclass correlation coefficient) in Whiting [12]. Six of the studies included in Pieper [17] assessed construct validity. No tests of validity were reported for any of the tools in Whiting [12] (although exploratory factor analysis was used to develop the content of AMSTAR). In addition, Pieper [17] reported data on the time to complete the assessment of each tool.

Of the three primary studies that evaluated RoB tools, two assessed the reliability and validity of AMSTAR and OQAQ [19, 34], one assessed the reliability and validity of the Rapid Appraisal Protocol internet Database (RAPiD) and the Quality and Applicability of Systematic Reviews of the National Center for the Dissemination of Rehabilitation Research (NCDRR) [34], and one reported the development and reliability of ROBIS [15] (Table 8). In addition, two of the three studies assessed the time to complete assessments [19, 34].

#### Assessment of risk of bias in studies evaluating methods

Both SRs [12, 17] were judged at low risk of bias, based on assessment using the ROBIS tool. Assessments for each domain are reported in Table 7. Of the four primary studies evaluating methods [15, 19, 34, 35]: (i) none referred to a study protocol or noted the existence of one, (ii) three used convenience samples as a method to select the sample of SRs to which the tool/measure was applied, (iii) the three studies that evaluated RoB tools either used a convenience sample, or provided no description, of the process for selecting raters who applied the tool and (iv) only one pre-specified hypotheses for testing of the validity of the measure [35] (Table 8).

## Discussion

In this paper, we present our developed framework of overview methods for the final steps in conducting an overview—assessment of the risk of bias in SRs and primary studies; synthesis, presentation and summary of the findings; and assessment of the certainty of evidence arising from the overview. We identified five stage II evaluation studies that mapped to the ‘assessment of the risk of bias in SRs and primary studies’ step of the framework and one study that mapped to the ‘synthesis, presentation and summary of the findings’ step. The evaluations included psychometric testing of tools to assess the risk of bias in SRs and development of a statistical measure to quantify overlap in primary studies across SRs. Results presented in this paper, in combination with our companion paper [10], provide a framework—the MOoR framework—of overview methods for all steps in the conduct of an overview. The framework makes explicit the large number of steps and methods that need to be considered when planning an overview and the unique decisions that need to be made as compared with a SR of primary studies. Here, we focus on issues pertinent to this second companion paper and present some overarching considerations.

### What this study adds to guidance and knowledge about overview methods

A key observation from our first paper, and aligned with conclusions of others [8, 9], was that there are important gaps in the guidance on the conduct of overviews [10]. Similar conclusions can be drawn from this paper, wherein guidance covers particular options, but not alternatives, and there is a lack of operational guidance for many methods. This is particularly pertinent for the step ‘assessment of the certainty of the evidence arising from the overview’, where GRADE methods (or equivalent) have yet to be developed for overviews. An exception was within the ‘assessment of risk of bias in SRs and primary studies’ step, where many tools for appraising or assessing the risk of bias in SRs have been developed, with psychometric evaluation for some tools, yielding at least some empirical evidence to underpin selection of tools. Detailed guidance on the applications of these tools has also been published.

The framework extends previous guidance on overviews methods [4, 39] through provision of a range of methods and options that might be used for each step. For most methods, we identified a lack of evaluation studies, indicating that there is limited evidence to inform methods decision-making in overviews. However, not all methods presented necessarily require evaluation. Theoretical considerations or poor face (or content) validity of a method may determine that it should not be used. For example, in the ‘assessment of risk of bias in SRs and primary studies’ step, an identified option (and one that has been used in some overviews) is to not report or assess RoB in the primary studies (2.1.4). Since the interpretation of evidence is highly dependent on limitations of primary studies within an SR, this option has little face validity.

A further extension to previous guidance is the linking of methods from our framework to address commonly arising challenges in overviews. This linking demonstrates that multiple methods are available for addressing each scenario, as illustrated in ‘Addressing common scenarios unique to overviews’ section using the example of the range of methods available for dealing with reviews that include overlapping primary studies.

### Strengths and limitations

The strengths and limitations described in the first paper in this series [10] are now briefly described here. The strengths of our research included (a) noting any deviations to our planned protocol [11], (b) using consistent language throughout the framework and an intuitive organising structure to group related methods and (c) drafting of the framework for each step by two authors independently. The limitations included the following: (a) the subjective nature of the research involving ‘translating’ descriptions of methods into a common language or standardised phrasing, (b) exclusion of articles that could have been of relevance to overviews (e.g. methods of indirect comparison and updating systematic reviews) and (c) difficulty in retrieving methods studies as methods collections are not routinely updated (for example, the Cochrane Methodology Register has not been updated since July 2012 [40]; and the Scientific Resource Center Methods library’s most recent article is from 2013).

An additional limitation is that new methods and methods evaluations may have been published since our last search (August 2016). However, we sought to identify methods that were missing from the literature (through inference) so the structure of the framework is unlikely to change. Given the sparsity of evidence about the performance of methods, any new evaluations will be an important addition to the evidence base but are unlikely to provide definitive evidence. One recent example is the publication of AMSTAR 2 [41]. While the development of AMSTAR 2 reflects an important advancement on the previous version of AMSTAR (extending to non-randomised studies and changing the response format), the tool will require application and further testing in overviews before its measurement properties can be fully established and compared to existing tools.

### Future research to refine and populate the framework and evidence map

Overview methods are evolving, and as methods are developed and evaluated, the evidence map can be further refined and populated. There are two related, but distinct streams of research here. The first stream relates to the development and application of methods. Substantial work is needed to provide detailed guidance for applying methods that have been advocated for use in overviews, in addition to developing new methods where gaps exist. The development of GRADE guidance for overviews is an important example where both methods development and detailed guidance is required.

The second stream of research involves methods evaluation. In our first paper, we suggested three domains against which the performance of overview methods should be evaluated: the validity and reliability of overview findings, the time and resources required to complete the overview, and the utility of the overview for decision-makers. For example, researchers could compare the statistical performance of different metrics to assess the degree of overlap, or different statistical methods to adjust for overlap in meta-analyses, using numerical simulation studies. A further area of research could include evaluation of different visual presentations of the range of summary results extracted from the constituent SRs. The framework will need to be refined, in response to methods development and evaluation. As mentioned in Paper 1, visual representation of an evidence map of overview methods will be useful when more evidence is available.

Furthermore, our framework and evidence map only focused on overviews of intervention reviews. The framework and evidence map could be extended to include methods for other types of overviews, such as overviews of diagnostic test accuracy reviews or prognostic reviews [42].

## Conclusions

A framework of methods for the final steps in conducting, interpreting and reporting overviews was developed, which in combination with our companion paper, provide a framework of overview methods—the MOoR framework—for all steps in the conduct of an overview. Evaluations of methods for overviews were identified and mapped to the framework. Many methods have been described for use in the latter steps in conducting an overview; however, evaluation and guidance for applying these methods is sparse. The exception is RoB assessment, for which a multitude of tools exist—several with sufficient evaluation and guidance to recommend their use. Evaluation of other methods is required to provide a comprehensive evidence map.

Further evaluation of methods for overviews will facilitate more informed methods decision-making. Results of this research may be used to identify and prioritise methods research, aid authors in the development of overview protocols and offer a basis for the development of reporting checklists.

## Additional files

Additional file 1: Main search strategies. (DOCX 16 kb)

Additional file 2: Purposive search strategy. (DOCX 16 kb)

Additional file 3: Characteristics of excluded studies. (DOCX 39 kb)

Additional file 4: Flowchart of purposive search strategy. (DOCX 42 kb)

Additional file 5: Table of reporting considerations. (PDF 102 kb)

## Abbreviations

AHRQ’s EPC: Agency for Healthcare Research and Quality s Evidence-based Practice Center
AMSTAR: A Measurement Tool to Assess Systematic Reviews
AQASR: Assessing the Quality and Applicability of Systematic Reviews
CA: Covered Area
CCA: Corrected Covered Area
CDSR: Cochrane Database of Systematic Reviews
CMIMG: Comparing Multiple Interventions Methods Group
HTA: Heath technology assessment
JBI: Joanna Briggs Institute
MA: Meta-analysis
MECIR: Methodological Expectations of Cochrane Intervention Reviews
NCDRR: Quality and Applicability of Systematic Reviews of the National Center for the Dissemination of Rehabilitation Research
OQAQ: Overview Quality Assessment Questionnaire
PICO: Population (P), intervention (I), comparison (C) and outcome (O)
PROSPERO: International Prospective Register of Systematic Reviews
RAPiD: Rapid Appraisal Protocol internet Database
RCT: Randomised controlled trial
RoB: Risk of bias
ROBIS: Risk of Bias In Systematic reviews
SRs: Systematic reviews

## References

[1] Caird J, Sutcliffe K, Kwan I, Dickson K, Thomas J (2015). Mediating policy-relevant evidence at speed: are systematic reviews of systematic reviews a useful approach?. Evid Policy.

[2] Lunny C, McKenzie JE, McDonald S (2016). Retrieval of overviews of systematic reviews in MEDLINE was improved by the development of an objectively derived and validated search strategy. J Clin Epidemiol.

[3] Pieper D, Buechter R, Jerinic P, Eikermann M (2012). Overviews of reviews often have limited rigor: a systematic review. J Clin Epidemiol.

[4] Becker LA, Oxman AD, JPT H, Green SE (2008). Chapter 22: Overviews of reviews. Cochrane Handbook for Systematic Reviews of Interventions.

[5] Bolland MJ, Grey A, Reid IR (2014). Differences in overlapping meta-analyses of vitamin D supplements and falls. J Clin Endocrinol Metab.

[6] Cooper H, Koenka AC (2012). The overview of reviews: unique challenges and opportunities when research syntheses are the principal elements of new integrative scholarship. Am Psychol.

[7] McKenzie JE, Brennan SE (2017). Overviews of systematic reviews: great promise, greater challenge. Syst Rev.

[8] Ballard M, Montgomery P (2017). Risk of bias in overviews of reviews: a scoping review of methodological guidance and four-item checklist. Res Synth Methods.

[9] Pollock M, Fernandes RM, Becker LA, Featherstone R, Hartling L (2016). What guidance is available for researchers conducting overviews of reviews of healthcare interventions? A scoping review and qualitative metasummary. Syst Rev.

[10] Lunny C, Brennan SE, McDonald S, McKenzie JE (2017). Toward a comprehensive evidence map of overview of systematic review methods: paper 1-purpose, eligibility, search and data extraction. Syst Rev.

[11] Lunny C, Brennan SE, McDonald S, McKenzie JE (2016). Evidence map of studies evaluating methods for conducting, interpreting and reporting overviews of systematic reviews of interventions: rationale and design. Syst Rev.

[12] Whiting P, Davies P, Savović J, Caldwell D, Churchill R (2013). Chapter 4. Phase 2: review of existing quality assessment tools for systematic reviews. Evidence to inform the development of ROBIS, a new tool to assess the risk of bias in systematic reviews.

[13] Mokkink LB, Terwee CB, Gibbons E, Stratford PW, Alonso J, Patrick DL, Knol DL, Bouter LM, de Vet HC (2010). Inter-rater agreement and reliability of the COSMIN (COnsensus-based Standards for the selection of health status Measurement Instruments) checklist. BMC Med Res Methodol.

[14] Mokkink LB, Terwee CB, Stratford PW, Alonso J, Patrick DL, Riphagen I, Knol DL, Bouter LM, de Vet HC (2009). Evaluation of the methodological quality of systematic reviews of health status measurement instruments. Qual Life Res.

[15] Whiting P, Savović J, Higgins JPT, Caldwell DM, Reeves BC, Shea B, Davies P, Kleijnen J, Churchill R, group R (2016). ROBIS: a new tool to assess risk of bias in systematic reviews was developed. J Clin Epidemiol.

[16] Bai A, Shukla VK, Bak G, Wells G (2012). Chapter 4: tools selected through QAT project. In: quality assessment tools project report.

[17] Pieper D, Buechter RB, Li L, Prediger B, Eikermann M. Systematic review found AMSTAR, but not R (evised)-AMSTAR, to have good measurement properties. J Clin Epidemiol. 2014; 10.1016/j.jclinepi.2014.12.009 25638457

[18] Whiting P, Davies P, Savović J, Caldwell D, Churchill R (2013). Chapter 5. Phase 3: review of studies that have used the AMSTAR tool. Evidence to inform the development of ROBIS, a new tool to assess the risk of bias in systematic reviews.

[19] Parmelli E, Banzi R, Fernandez Del Rio MDP, Minozzi S, Moja L, Pecoraro V, Liberati A: Using AMSTAR to assess the methodological quality of systematic reviews: an external validation study. Poster presentation at the 19th Cochrane Colloquium; 2011 Oct 19-22; Madrid, Spain [abstract]. In Cochrane Database Syst Rev, Supplement, vol. Suppl. pp. 139; 2011:139.

[20] Popovich I, Windsor B, Jordan V, Showell M, Shea B, Farquhar CM (2012). Methodological quality of systematic reviews in subfertility: a comparison of two different approaches. PLoS ONE.

[21] Schmitter M, Sterzenbach G, Faggion CM, Krastl G (2013). A flood tide of systematic reviews on endodontic posts: methodological assessment using of R-AMSTAR. Clin Oral Investig.

[22] Shea BJ, Grimshaw JM, Wells GA, Boers M, Andersson N, Hamel C, Porter AC, Tugwell P, Moher D, Bouter LM (2007). Development of AMSTAR: a measurement tool to assess the methodological quality of systematic reviews. BMC Med Res Methodol.

[23] Shea BJ, Hamel C, Wells GA, Bouter LM, Kristjansson E, Grimshaw J, Henry DA, Boers M (2009). AMSTAR is a reliable and valid measurement tool to assess the methodological quality of systematic reviews. J Clin Epidemiol.

[24] Robinson KA, Chou R, Berkman ND, Newberry SJ, Fu R, Hartling L, Dryden D, Butler M, Foisy M, Anderson J (2016). Twelve recommendations for integrating existing systematic reviews into new reviews: EPC guidance. J Clin Epidemiol.

[25] Ryan RE, Kaufman CA, Hill SJ (2009). Building blocks for meta-synthesis: data integration tables for summarising, mapping, and synthesising evidence on interventions for communicating with health consumers. BMC Med Res Methodol.

[26] Thomson D, Russell K, Becker L, Klassen TP, Hartling L (2010). The evolution of a new publication type: steps and challenges of producing overviews of reviews. Res Syn Method.

[27] Dobbins M (2016). Health Evidence (TM): a public health knowledge repository disseminating evidence to decision makers. Euro J Public Health.

[28] Oxman AD, Guyatt GH (1991). Validation of an index of the quality of review articles. J Clin Epidemiol.

[29] Jadad AR, Cook DJ, Browman GP (1997). A guide to interpreting discordant systematic reviews. Cmaj.

[30] Guyatt GH, Oxman AD, Vist GE, Kunz R, Falck-Ytter Y, Alonso-Coello P, Schunemann HJ (2008). GRADE: an emerging consensus on rating quality of evidence and strength of recommendations. Bmj.

[31] Pollock A, Farmer SE, Brady MC, Langhorne P, Mead GE, Mehrholz J, van Wijck F, Wiffen PJ. An algorithm was developed to assign GRADE levels of evidence to comparisons within systematic reviews. J Clin Epidemiol. 2015; 10.1016/j.jclinepi.2015.08.013 PMC4742519 26341023

[32] Murad MH, Mustafa R, Morgan R, Sultan S, Falck-Ytter Y, Dahm P (2016). Rating the quality of evidence is by necessity a matter of judgment. J Clin Epidemiol.

[33] Berkman ND, Lohr KN, Ansari MT, Balk EM, Kane R, McDonagh M, Morton SC, Viswanathan M, Bass EB, Butler M (2015). Grading the strength of a body of evidence when assessing health care interventions: an EPC update. J Clin Epidemiol.

[34] Pieper D, Mathes T, Eikermann M (2014). Impact of choice of quality appraisal tool for systematic reviews in overviews. J Evid Based Med.

[35] Pieper D, Antoine SL, Mathes T, Neugebauer EA, Eikermann M (2014). Systematic review finds overlapping reviews were not mentioned in every other overview. J Clin Epidemiol.

[36] Kung J, Chiappelli F, Cajulis OO, Avezova R, Kossan G, Chew L (2010). From systematic reviews to clinical recommendations for evidence-based health care: validation of revised assessment of multiple systematic reviews (R-AMSTAR) for grading of clinical relevance. Open Dent J.

[37] Shea BJ, Bouter LM, Peterson J, Boers M, Andersson N, Ortiz Z, Ramsay T, Bai A, Shukla VK, Grimshaw JM (2007). External validation of a measurement tool to assess systematic reviews (AMSTAR). PLoS One.

[38] Higgins JPT, Lane PW, Anagnostelis B, Anzures-Cabrera J, Baker NF, Cappelleri JC, Haughie S, Hollis S, Lewis SC, Moneuse P, Whitehead A (2013). A tool to assess the quality of a meta-analysis. Res Synth Methods.

[39] Joanna Briggs Institute (2014). Methodology for JBI Umbrella Reviews.

[40] Cochrane Methods Group. About the Cochrane Methodology Register: Cochrane; 2012. http://www.cochranelibrary.com/help/the-cochrane-methodology-register-july-issue-2012.html

[41] Shea BJ, Reeves BC, Wells G, Thuku M, Hamel C, Moran J, Moher D, Tugwell P, Welch V, Kristjansson E, Henry DA (2017). AMSTAR 2: a critical appraisal tool for systematic reviews that include randomised or non-randomised studies of healthcare interventions, or both. BMJ.

[42] Hunt H, Pollock A, Campbell P, Estcourt L, Brunton G (2018). An introduction to overviews of reviews: planning a relevant research question and objective for an overview. Syst Rev.

[43] Baker PRA, Costello JT, Dobbins M, Waters EB (2014). The benefits and challenges of conducting an overview of systematic reviews in public health: a focus on physical activity. J Publ Health.

[44] Brunton G, Thomas J, Paraskeva N, Caird J, Rumsey N. Putting the issues on the table: summarising outcomes from reviews of reviews to inform health policy. In: Cochrane Colloquium. Québec City; 2006.

[45] Büchter R, Pieper D. How do authors of Cochrane Overviews deal with conflicts of interest relating to their own systematic reviews? In: Cochrane Colloquium. Vienna; 2015.

[46] Chen YF, Hemming K, Chilton PJ, Gupta KK, Altman DG, Lilford RJ (2014). Scientific hypotheses can be tested by comparing the effects of one treatment over many diseases in a systematic review. J Clin Epidemiol.

[47] CMIMG C (2012). Review Type & Methodological Considerations --Background Paper for the First Part of the Paris CMIMG Discussion.

[48] Crick K, Wingert A, Williams K, Fernandes RM, Thomson D, Hartling L (2015). An evaluation of harvest plots to display results of meta-analyses in overviews of reviews: a cross-sectional study. BMC Med Res Methodol.

[49] Flodgren G, Shepperd S, Eccles M. Challenges facing reviewers preparing overviews of reviews (P2A194). In: Cochrane Colloquium. Madrid; 2011.

[50] Foisy M, Becker LA, Chalmers JR, Boyle RJ, Simpson EL, Williams HC. Mixing with the ‘unclean’: including non-Cochrane reviews alongside Cochrane reviews in overviews of reviews (P2A157). In: Cochrane Colloquium. Madrid; 2011.

[51] Foisy MFR, Dryden DM, Hartling L (2014). Grading the quality of evidence in existing systematic reviews: challenges and considerations. 22nd Cochrane Colloquium.

[52] Foisy M, Hartling L. Challenges and considerations involved in using AMSTAR in overviews of reviews. In: Cochrane Colloquium. Hyderabad; 2014.

[53] Hartling L, Chisholm A, Thomson D, Dryden DM (2012). A descriptive analysis of overviews of reviews published between 2000 and 2011. PloS one.

[54] Hartling LDD, Vandermeer B, Fernandes R. Generating empirical evidence to support methods for overviews of reviews. In: Cochrane Colloquium. Quebec City; 2013.

[55] Hartling L, Vandermeer B, Fernandes RM (2014). Systematic reviews, overviews of reviews and comparative effectiveness reviews: a discussion of approaches to knowledge synthesis. Evid Based Child Health.

[56] Hemming K, Bowater RJ, Lilford RJ (2012). Pooling systematic reviews of systematic reviews: a Bayesian panoramic meta-analysis. Stat Med.

[57] Ioannidis JPA (2009). Integration of evidence from multiple meta-analyses: a primer on umbrella reviews, treatment networks and multiple treatments meta-analyses. CMAJ.

[58] James BM, Baker PRA, Costello JT, Francis DP. Informing methods for preparing public health overviews of reviews: a comparison of public health overviews with Cochrane Overviews published between 1999 and 2014. In: Cochrane Colloquium. Hyderabad; 2014.

[59] Aromataris E, Fernandez R, Godfrey CM, Holly C, Khalil H, Tungpunkom P (2015). Summarizing systematic reviews: methodological development, conduct and reporting of an umbrella review approach. Int J Evid Based Healthc.

[60] Kovacs FM, Urrutia G, Alarcon JD (2014). “Overviews” should meet the methodological standards of systematic reviews. Eur Spine J.

[61] Kramer S, Langendam M, Elbers R, Scholten R, Hooft L. Preparing an overview of reviews: lessons learned. Poster. In: Cochrane Colloquium; 2009 Oct 11-14. Singapore; 2009.

[62] Li LM, Tian JT, Tian H, Sun R, Liu Y, Yang K (2012). Quality and transparency of overviews of systematic reviews. J Evid-Based Med.

[63] Moja L, Fernandez del Rio MP, Banzi R, Cusi C, D'Amico R, Liberati A, Lodi G, Lucenteforte E, Minozzi S, Pecoraro V (2012). Multiple systematic reviews: methods for assessing discordances of results. Intern Emerg Med.

[64] O'Mara AJ, Jamal F, Parry W, Lorenc T, Cooper C. Guidelines for conducting and reporting reviews of reviews: dealing with topic relevances and double-counting. Poster presentation at the 19th Cochrane Colloquium; 2011 Oct 19-22; Madrid, Spain [abstract]. In Cochrane Database Syst Rev, Supplement, issue CD000003. 2011. p. 101. Available at: https://cmr.cochrane.org/?CRGReportID=16702.

[65] Büchter R, Pieper D, Jerinic P (2011). Overviews of systematic reviews often do not assess methodological quality of included reviews. Poster. 19th Cochrane Colloquium.

[66] Pieper DA, Morfeld S-L, Mathes J-C, Mathes T, Eikermann M (2014). Methodological approaches in conducting overviews: current state in HTA agencies. Res Syn Method.

[67] JPT H, Green S, editors. Cochrane Handbook for Systematic Reviews of Interventions Version 5.1.0 [updated March 2011]. In: : The Cochrane Collaboration. p. 2011. Available from http://handbook.cochrane.org.

[68] Pieper D, Antoine S, Neugebauer EA, Eikermann M (2014). Up-to-dateness of reviews is often neglected in overviews: a systematic review. J Clin Epidemiol.

[69] Robinson KA, Chou R, Berkman ND, Newberry SJ, Fu R, Hartling L, Dryden D, Butler M, Foisy M, Anderson J, Motu’apuaka ML, Relevo R, Guise JM, Chang S (2008). Integrating bodies of evidence: existing systematic reviews and primary studies. Methods Guide for Effectiveness and Comparative Effectiveness Reviews.

[70] Robinson KA, Whitlock EP, O'Neil ME, Anderson JK, Hartling L, Dryden DM, Butler M, Newberry SJ, McPheeters M, Berkman ND (2014). Integration of existing systematic reviews. In Research White Paper (Prepared by the Scientific Resource Center under Contract No 290-2012-00004-C).

[71] White CM, Ip S, McPheeters MC, Tim S, Chou R, Lohr KN, Robinson K, McDonald K, Whitlock EP (2009). Using existing systematic reviews to replace de novo processes in conducting comparative effectiveness reviews. In Methods Guide for Comparative Effectiveness Reviews.

[72] Whitlock EP, Lin JS, Chou R, Shekelle P, Robinson KA (2008). Using existing systematic reviews in complex systematic reviews. Ann Intern Med.

[73] Salanti G, Becker L, Caldwell D, Churchill R, Higgins J, Li T, Schmid C (2011). Evolution of Cochrane Intervention Reviews and Overviews of Reviews to better accommodate comparisons among multiple interventions. Report from a meeting of the Cochrane Comparing Multiple Interventions Methods Groups: Cochrane Comparing Multiple Interventions Methods Groups.

[74] Schmidt FL, Oh IS (2013). Methods for second order meta-analysis and illustrative applications. Organ Behav Hum Decis Process.

[75] Silva V, Grande AJ, Carvalho AP, Martimbianco AL, Riera R (2015). Overview of systematic reviews - a new type of study. Part II. Sao Paulo Med J.

[76] Singh JP (2012). Development of the Metareview Assessment of Reporting Quality (MARQ) Checklist. Rev Fac Med Univ Nac Colomb.

[77] Smith V, Devane D, Begley CM, Clarke M (2011). Methodology in conducting a systematic review of systematic reviews of healthcare interventions. BMC Med Res Methodol.

[78] Tang LL, Caudy M, Taxman F (2013). A statistical method for synthesizing meta-analyses. Comput Math Methods Med.

[79] Thomson D, Foisy M, Oleszczuk M, Wingert A, Chisholm A, Hartling L (2013). Overview of reviews in child health: evidence synthesis and the knowledge base for a specific population. Evidence Based Child Health.

[80] Wagner S, White M, Schultz I, Iverson R, Hsu V, McGuire L, Schultz W. Assessing a systematic review of systematic reviews: developing a criteria. In: Innovation in worker health and safety: Annual Conference, Canadian Association for Research on Work and Health, June 1-2, 2012. Vancouver; 2012. https://www.wwdpi.org/SiteCollectionDocuments/CIRPD-Research/CARWH2012/P3_MethodologicalCriteria.pdf.

[81] McMaster University (2011). Health systems evidence.

[82] Patnode CD, Henderson JT, Thompson JH, Senger CA, Fortmann SP, Whitlock EP (2015). Behavioral counseling and pharmacotherapy interventions for tobacco cessation in adults, including pregnant women: a review of reviews for the U.S. Preventive Services Task Force. Ann Intern Med.

[83] Unit PHR (2006). Critical Appraisal Skills Programme (CASP). 10 questions to help you make sense of reviews.

[84] FOCUS (2001). FOCUS critical appraisal tool.

[85] Beck CT (1997). Use of meta-analysis as a teaching strategy in nursing research courses. J Nurs Educ.

[86] (NHMRC) National Health and Medical Research Council. How to review the evidence: assessment and application of scientific evidence. http://www.nhmrc.gov.au/guidelines/publications/cp69. Canberra; 2000.

[87] (SIGN) Scottish Intercollegiate Guidelines Network HIS, SIGN 50. Methodology checklist 1: systematic reviews and meta-analyses. Edinburgh: Scottish Intercollegiate Guidelines Network, Healthcare Improvement Scotland; 2009. Available at: http://www.sign.ac.uk/checklists-and-notes.html.

[88] Joanna Briggs Institute (2006). RAPid: Rapid Appraisal protocol internet database.

[89] Assendelft WJ, Koes BW, Knipschild PG, Bouter LM (1995). The relationship between methodological quality and conclusions in reviews of spinal manipulation. JAMA.

[90] Auperin A, Pignon JP, Poynard T (1997). Review article: critical review of meta-analyses of randomized clinical trials in hepatogastroenterology. Aliment Pharmacol Ther.

[91] Crombie IK (1996). The pocket guide to critical appraisal: a handbook for health care professionals.

[92] Geller NL, Proschan M (1996). Meta-analysis of clinical trials: a consumer’s guide. J Biopharm Stat.

[93] Glenny A, Esposito M, Coulthard P, Worthington H (2003). The assessment of systematic reviews in dentistry. Eur J Oral Sci.

[94] Greenhalgh T (1997). Papers that summarise other papers (systematic reviews and meta-analyses). Bmj.

[95] Ho RC, Ong HS, Kudva KG, Cheung MW, Mak A (2010). How to critically appraise and apply meta-analyses in clinical practice. Int J Rheum Dis.

[96] Irwig L, Tosteson AN, Gatsonis C, Lau J, Colditz G, Chalmers TC, Mosteller F (1994). Guidelines for meta-analyses evaluating diagnostic tests. Ann Intern Med.

[97] Knox EM, Thangaratinam S, Kilby MD, Khan KS (2009). A review of the methodological features of systematic reviews in fetal medicine. Eur J Obstet Gynecol Reprod Biol.

[98] Li T, Vedula SS, Scherer R, Dickersin K (2012). What comparative effectiveness research is needed? A framework for using guidelines and systematic reviews to identify evidence gaps and research priorities. Ann Intern Med.

[99] Light RJ, Pillemer DB (1984). The science of reviewing research.

[100] Lundh A, Sismondo S, Lexchin J, Busuioc O, Bero L. Industry sponsorship and research outcome. Cochrane Database Syst Rev. 2015;12 10.1002/14651858.MR000033.pub2 23235689

[101] Mailis A, Taenzer P (2012). Evidence-based guideline for neuropathic pain interventional treatments: spinal cord stimulation, intravenous infusions, epidural injections and nerve blocks. Pain Res Manag.

[102] Minelli C, Thompson JR, Abrams KR, Thakkinstian A, Attia J (2009). The quality of meta-analyses of genetic association studies: a review with recommendations. Am J Epidemiol.

[103] Mulrow CD (1987). The medical review article: state of the science. Ann Intern Med.

[104] Nony P, Cucherat M, Haugh MC, Boissel JP (1995). Critical reading of the meta-analysis of clinical trials. Therapie.

[105] Oxman AD, Guyatt GH (1988). Guidelines for reading literature reviews. Cmaj.

[106] Oxman AD, Cook DJ, Guyatt GH (1994). Users’ guides to the medical literature. VI. How to use an overview. Evidence-Based Medicine Working Group. JAMA.

[107] Oxman AD (1994). Checklists for review articles. BMJ.

[108] Philibert A, Loyce C, Makowski D (2012). Assessment of the quality of meta-analysis in agronomy. Agric Ecosyst Environ.

[109] Sacks HS, Berrier J, Reitman D, Ancona-Berk V, Chalmers TC (1987). Meta-analyses of randomized controlled trials. N Engl J Med.

[110] Santaguida P, Oremus M, Walker K, Wishart LR, Siegel KL, Raina P (2012). Systematic reviews identify important methodological flaws in stroke rehabilitation therapy primary studies: review of reviews. J Clin Epidemiol.

[111] Shamliyan T, Kane RL, Jansen S (2010). Quality of systematic reviews of observational nontherapeutic studies. Prev Chronic Dis.

[112] Sheikh L, Johnston S, Thangaratinam S, Kilby MD, Khan KS (2007). A review of the methodological features of systematic reviews in maternal medicine. BMC Med.

[113] Smith AF (1997). An analysis of review articles published in four anaesthesia journals. Can J Anaesth.

[114] Thacker SB, Peterson HB, Stroup DF (1996). Metaanalysis for the obstetrician-gynecologist. Am J Obstet Gynecol.

[115] Wilson A, Henry DA (1992). Meta-analysis. Part 2: assessing the quality of published meta-analyses. Med J Aust.

[116] Zambon M, Biondi-Zoccai G, Bignami E, Ruggeri L, Zangrillo A, Landoni G (2012). A comprehensive appraisal of meta-analyses focusing on nonsurgical treatments aimed at decreasing perioperative mortality or major cardiac complications. J Anesth.

[117] Task Force on Systematic Review and Guidelines (2011). Assessing the quality and applicability of systematic reviews (AQASR).

[118] Higgins JPT, Lasserson T, Chandler J, Tovey D, Churchill R. Methodological Expectations of Cochrane Intervention Reviews. Cochrane: London, Version 1.05, 2018. Available at: https://community.cochrane.org/mecir-manual.

