# Supplementary material for: Toward a comprehensive evidence map of overview of systematic review methods: paper 2—risk of bias assessment; synthesis, presentation and summary of the findings; and assessment of the certainty of the evidence
Source: Syst Rev. 2018 Oct 12;7:159. doi: 10.1186/s13643-018-0784-8 (PMC6186052; doi:10.1186/s13643-018-0784-8)
Supplement: Supplementary file 2 — Purposive search strategy. (DOCX 16 kb) [file 13643_2018_784_MOESM2_ESM.docx]

**Additional File 2**

**Purposive search strategies (January 2013-August 2016)**

We used the search strategy by Whiting et al. {Whiting, 2013 #2} to retrieve quality assessment or critical appraisal tools aimed at assessing systematic reviews or meta-analyses in MEDLINE.

**Ovid MEDLINE** 1946 to August 2016

1 "Review Literature as Topic"/ (6589)

2 meta-analysis/ (72836)

3 meta-analysis as topic/ (15341)

4 systematic review$.tw. (70220)

5 (meta-analys$ or metaanalys$).tw. (82084)

6 or/1-5 (152816)

7 Checklist/ (3806)

8 Quality Control/ (44280)

9 Guidelines as Topic/ (34856)

10 Total Quality Management/ (12154)

11 Reference Standards/ (38377)

12 or/7-11 (128318)

13 ((tool or tools or instrument$ or checklist$ or check list$ or scale or scales) and (quality or methodolog$ or method or methods)).ti. (6902)

14 (quality adj10 (score or scores or scoring or rating or rate) adj5 (methodolog$ or method or methods)).tw. (1280)

15 (guideline$ and (quality or methodolog$ or method or methods)).ti. (2163)

16 ((assess$ or apprais$ or critical$) adj3 (systematic review$ or meta-analys$ or metaanalys$)).ti. (611)

17 ((score or scores or scoring or rating or rate) and (quality or methodolog$ or method or methods)).ti. (5168)

18 ((quality or methodology) adj3 (review or meta-analys$ or metaanalys$) adj3 (assess$ or method$)).tw. (2284)

19 (quality adj3 article$).tw. (1589)

20 (critical$ adj2 (apprais$ or evaluat$)).tw. (17141)

21 ((apprais$ or evaluat$) adj3 (systematic review$ or meta-analys$ or metaanalys$)).tw. (4364)

22 (guideline$ adj3 (systematic review$ or meta-analys$ or metaanalys$)).tw. (1397)

23 or/13-22 (41243)

24 12 or 23 (167287)

25 Publication Bias/ (3741)

26 exp "bias (epidemiology)"/ (57971)

27 "Reproducibility of Results"/ (324579)

28 "Review Literature as Topic"/ (6589)

29 meta-analysis as topic/ (15341)

30 (bias adj3 (systematic review$ or meta-analys$ or metaanalys$)).tw. (620)

31 ((quality or bias or methodolog$) adj3 (systematic review$ or meta-analys$ or metaanalys$)).tw. (2924)

32 or/25-31 (385308)

33 6 and 24 and 32 (3479)

34 (201305$ or 201306$ or 201307$ or 201308$ or 201209$ or 20131$).ed. (378315)

35 33 and 34 (124)

36 (2014$ or 2015$ or 201601$ or 201602$ or 201603$ or 201604$ or 201605$ or 201606$ or 201607$ or 201608$).ed. (2368353)

37 33 and 36 (1108)

38 35 or 37 (1232)

39 limit 38 to (English language and humans (1097)

**Meth4ReSyn library aug 26 2016**

<http://www.citeulike.org/user/Meth4ReSyn/tag/assessment_of_review_practice>

n=26
<http://www.citeulike.org/user/Meth4ReSyn/tag/review_quality>

n=17

total 43 (10 are duplicates)

**Scientific Resource Center Methods library of the AHRQ Effective Health Care Program**

(tag:quality-assessment) && "reviews" n=42

(tag:quality-assessment) n-117

**Cochrane Colloquium abstracts (2013-2016, limit posters)**

<http://abstracts.cochrane.org>

("quality assessment*" and reviews) (45)

("assessment tool" and reviews) (13)

("quality of the evidence" and reviews) (11)

("strength of the evidence" and reviews) (3)

("GRADE" and overviews) (13)
