# Supplementary material for: Toward a comprehensive evidence map of overview of systematic review methods: paper 2—risk of bias assessment; synthesis, presentation and summary of the findings; and assessment of the certainty of the evidence
Source: Syst Rev. 2018 Oct 12;7:159. doi: 10.1186/s13643-018-0784-8 (PMC6186052; doi:10.1186/s13643-018-0784-8)
Supplement: Supplementary file 3 — Characteristics of excluded studies. (DOCX 39 kb) [file 13643_2018_784_MOESM3_ESM.docx]

**Additional file 3**

**Characteristics of excluded studies**

| **Citation** | **Reason for exclusion** |
| --- | --- |
| Adams 2000 [1]* | Study not examining methods in a cross-section or cohort of overviews – does not discuss methods |
| Bai 2012 [2]** | Excluded due to overlap in inclusion with the included SR by Whiting 2013 [3]*** |
| Bekkering 2013 [4]* | Study not examining methods in a cross-section or cohort of overviews – not a cohort of overviews |
| Bjordal 2008 [5] | Study not examining methods in a cross-section or cohort of overviews – does not discuss methods |
| Bramer 2013 [6] | Study not examining methods in a cross-section or cohort of overviews – not a cohort of overviews |
| Costa 2013 [7]* | Study not examining methods in a cross-section or cohort of overviews – does not discuss methods |
| Delgado-Rodriguez 2006 [8] | Study not examining methods in a cross-section or cohort of overviews – not a cohort of overviews |
| Elliott 2004 [9] | Study not examining methods in a cross-section or cohort of overviews – not a cohort of overviews |
| Holt 2013 [10] | Study not examining methods in a cross-section or cohort of overviews – does not discuss methods |
| Hyde 2006 [11]* | Study not examining methods in a cross-section or cohort of overviews – not a cohort of overviews |
| Jefferson 2000 [12]* | Study not examining methods in a cross-section or cohort of overviews – not a cohort of overviews |
| Levin 2009 [13] | Study not examining methods in a cross-section or cohort of overviews – does not discuss methods |
| Lopez 2010 [14]* | Study not examining methods in a cross-section or cohort of overviews |
| Martinez-Zapata 2011 [15]** | Not a study that evaluated methods – not a cohort of overviews |
| Pantoja 2015 [16]* | Study not examining methods in a cross-section or cohort of overviews |
| Popovich 2012 [17]** | Excluded due to overlap in inclusion with the included SR by Whiting 2013 [3]*** |
| Rada 2013 [18] | Study not examining methods in a cross-section or cohort of overviews – not a cohort of overviews |
| Rojas 2011 [19] | Study not examining methods in a cross-section or cohort of overviews |
| Ryan 2005 [20]* | Study not examining methods in a cross-section or cohort of overviews |
| Santaguida 2013 [21] | Study not examining methods in a cross-section or cohort of overviews – not a cohort of overviews |
| Schmitter 2013 [22]** | Excluded due to overlap in inclusion with the included SR by Whiting 2013 [3]*** |
| Shea 2007 [23, 24]** | Excluded due to overlap in inclusion with the included SR by Whiting 2013 [3]*** |
| Shea 2009 [25]** | Excluded due to overlap in inclusion with the included SR by Whiting 2013 [3]*** |
| Tanjong-Ghogomu 2010 [26]* | Study not examining methods in a cross-section or cohort of overviews |
| Thomson 2014 [27] | Study not examining methods in a cross-section or cohort of overviews – does not discuss methods |
| Wang 2012 [28]* | Study not examining methods in a cross-section or cohort of overviews – not a cohort of overviews |

* Conference poster

** Stage II study

*** Included in our stage II analysis

**References**

1. Adams C, Dooley, G., Jefferson, T., Lancaster, T.: Overviews - the way forward for the collaboration? *Cochrane Colloquium*. Cape Town, South Africa; 2000.

2. Bai, A., Shukla, VK., Bak, G., Wells, G.: Chapter 4: Tools selected through QAT project. Quality assessment tools project report. Ottawa, Ontario, Canada: Canadian Agency for Drugs and Technologies in Health; 2012.

3. Whiting, P., Davies, P., Savović, J., Caldwell, D., Churchill, R.: Chapter 5. Phase 3: Review of studies that have used the AMSTAR tool. Evidence to inform the development of ROBIS, a new tool to assess the risk of bias in systematic reviews, <http://www.robis-tool.info> [Accessed 201802/24]. 2013.

4. Bekkering, G.E.: Is there a potential of umbrella reviews to inform guideline development? *Cochrane Colloquium*. Québec City, Canada; 2013.

5. Bjordal, J.M., Klovning, A., Lopes-Martins, R.A., Roland, P.D., Joensen, J., Slordal, L.: Overviews and systematic reviews on low back pain. *Ann Intern Med.* 2008, 148:789-790; author reply 791-782.

6. Bramer, W.M., Giustini, D., Kramer, B.M., Anderson, P.: The comparative recall of Google Scholar versus PubMed in identical searches for biomedical systematic reviews: a review of searches used in systematic reviews. *Syst Rev* 2013, 2:115.

7. Costa, M.B., Porfírio, G.J.M., Silva, V., Grande, A., Torres, M.F.S., Carvalho, M.R., Fioretti, B., Riera, R., Torloni, M.R.: Profile of overviews published by the Cochrane Library. *Cochrane Colloquium*. Quebec City, Canada; 2013.

8. Delgado-Rodriguez, M.: Systematic reviews of meta-analyses: applications and limitations. *J Epi & Comm Health*. 2006, 60:90-92.

9. Elliott, L., Crombie, I.K., Irvine, L., Cantrell, J., Taylor, J.: The effectiveness of public health nursing: the problems and solutions in carrying out a review of systematic reviews. *J Adv Nursing.* 2004, 45:117-125.

10. Holt, R.I.: A review of reviews: a virtual issue. *Diabetes Obes Metab.* 2013, 15:1-2.

11. Hyde, C.: Identifying systematic reviews on related topics: can we do more to help readers? *Cochrane Colloquium*. Dublin, Ireland; 2006.

12. Jefferson, T., Demicheli,, V., Jefferson, T., Middleton, P., Wager, E.: An overview of the effects of peer review on the assessment of scientific submissions to journals and grant-giving bodies methodological issues. *Symposium on Systematic Reviews: Beyond the Basics*. Oxford, UK; 2000.

13. Levin, R.F.: Reviews, systematic reviews, overviews: "What's it all about, Cochrane"? *Res Theory Nurs Pract.* 2009, 23:256-258.

14. Lopez, L., Grimes, D., Manion, C.: When it rains: synthesizing umbrella reviews of educational interventions In *Joint Colloquium of The Cochrane and Campbell Collaborations*. Keystone, USA; 2010.

15. Martinez-Zapata, M.J., Rigau, D., Selva, A., Gich, I., Bonfill, X.: Applicability of R-AMSTAR instrument to appraise systematic reviews. *Cochrane Colloquium*. Madrid, Spain; 2011.

16. Pantoja, T., Opiyo, N., Ciaponni, A., Herrera, C., Lewin, S., Oxman, A., Paulsen, E., Rada, G., Wiysonge, C. : Strategies for improving health systems in low-income countries: lessons learnt from four overviews of systematic reviews of health systems interventions. *Cochrane Colloquium.* Vienna, Austria; 2015

17. Popovich I, Windsor B, Jordan V, Showell M, Shea B, Farquhar CM: Methodological quality of systematic reviews in subfertility: a comparison of two different approaches. *PLoS ONE.* 2012, 7:e50403.

18. Rada, G., Perez, D., Capurro, D.: Epistemonikos: a free, relational, collaborative, multilingual database of health evidence. *Stud Health Technol Inform.* 2013, 192:486-490.

19. Rojas, M., Lozano, J., Sola, I., Bonfill, X.: Incorporating the GRADE approach in overviews of systematic reviews: an example from an overview in neonatal respiratory care. *Cochrane Colloquium*. Madrid, Spain; 2011.

20. Ryan, R., Hill, S.: Evidence overviews: prioritising areas for overviews of systematic reviews of interventions for effective communication with and participation by consumers. *Cochrane Colloquium*. Melbourne, Australia; 2005.

21. Santaguida, P.L., Keshavarz, H., Carlesso, L.C., Lomotan, M., Gross, A., MacDermid, J.C., Walton, D.M., ICON Working Group: Suppl 4: A Description of the Methodology Used in an Overview of Reviews to Evaluate Evidence on the Treatment, Harms, Diagnosis/Classification, Prognosis and Outcomes Used in the Management of Neck Pain. *Open Orthopaed J.* 2013, 7:461.

22. Schmitter, M., Sterzenbach, G., Faggion, Jr. C., Krastl, G.: A flood tide of systematic reviews on endodontic posts: methodological assessment using of R-AMSTAR. *Clin oral investig.* 2013, 17:1287-1294.

23. Shea, B.J., Wells, G., Boers, M., Hamel, C., Porter, A., Moher, D., Tugwell, P., Bouter, L.: Advancement of the methodological quality through a measurement tool to assess reviews: AMSTAR. *14th Cochrane Colloquium.* Dublin, Ireland; 2006

24. Shea, B.J., Grimshaw, J.M., Wells, G.A., Boers, M., Andersson, N., Hamel, C., Porter, A.C., Tugwell, P., Moher, D., Bouter, L.M.: Development of AMSTAR: a measurement tool to assess the methodological quality of systematic reviews. *BMC Med Res Method*, 2007, 7:10.

25. Shea, B.J., Hamel, C., Wells, G.A., Bouter, L.M., Kristjansson, E., Grimshaw, J., Henry, D.A., Boers, M.: AMSTAR is a reliable and valid measurement tool to assess the methodological quality of systematic reviews. *J Clin Epi* 2009, 62:1013-1020.

26. Tanjong-Ghogomu E, Singh, J., Christensen, R., Wells, G., Suarez-Almazor, M., Buchbinder, R., Lopez-Olivo, A., Tugwell, P.: Overviews of reviews –methodological considerations of the Biologics for rheumatoid arthritis Cochrane overview. *Cochrane Colloquium*. Keystone, USA; 2010.

27. Thomson, D.: Evidence synthesis in child health: overviews of reviews. *Evid Based Child Health* 2014, 9:1-2.

28. Wang X, Lindsley, K., Li, T.: Is there agreement in outcomes among Cochrane reviews to support ‘Overviews’ of reviews? a case study within the Cochrane Eyes and Vision Group (CEVG). *Cochrane Colloquium*. Auckland, New Zealand; 2012.
