# Supplementary figures and images for: Toward a comprehensive evidence map of overview of systematic review methods: paper 2—risk of bias assessment; synthesis, presentation and summary of the findings; and assessment of the certainty of the evidence
Source: Syst Rev. 2018 Oct 12;7:159. doi: 10.1186/s13643-018-0784-8 (PMC6186052; doi:10.1186/s13643-018-0784-8)

**Additional file 4**

**Figure 4: Flowchart of stage II purposive search**


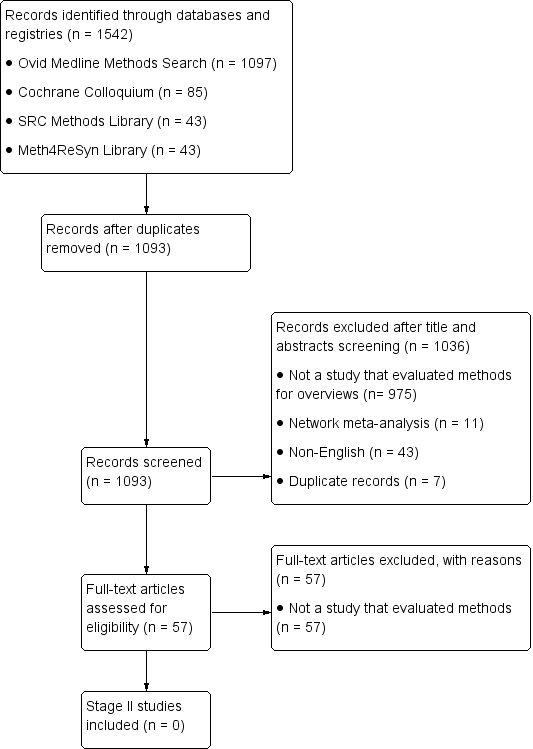

Supplement: Supplementary file 4 — Flowchart of purposive search strategy. (DOCX 42 kb) [file 13643_2018_784_MOESM4_ESM.docx]
