# Supplementary material for: Toward a comprehensive evidence map of overview of systematic review methods: paper 2—risk of bias assessment; synthesis, presentation and summary of the findings; and assessment of the certainty of the evidence
Source: Syst Rev. 2018 Oct 12;7:159. doi: 10.1186/s13643-018-0784-8 (PMC6186052; doi:10.1186/s13643-018-0784-8)
Supplement: Supplementary file 5 — Table of reporting considerations. (PDF 102 kb) [file 13643_2018_784_MOESM5_ESM.pdf]

Additional file 5
Table of reporting considerations

We identified several tools to assess the reporting quality of overviews, although none of these was explicitly developed as consensus-based reporting guidelines (1-3). Hartling 2012 (1) compared the applicability of the Methodological Expectations of Cochrane Intervention Reviews (MECIR) standards developed by Cochrane (4) to proposed methods for reporting in overviews. Li 2012 (2) developed a quality of conduct and reporting checklist based on the Cochrane Handbook (5), PRISMA (6) and OQAQ (7) tools for systematic reviews. Singh 2012 (3) developed a reporting tool for meta-reviews 'using previously published checklists' without stating which checklists they referenced when developing their tool. It was unclear whether this tool was intended for any overview of reviews, or only for reviews examining the methodological conduct of systematic reviews.

Step	Sub-step Methods/approaches	Sources
**1.0 Assessment of risk of bias in SRs and primary studies**		
	1.1 Report methods and the tool used to assess risk of bias (RoB) in SRs	Becker & Oxman 2008 (8); Cooper 2012 (9); Hartling 2012 (1); JBI 2014 (10, 11); Li 2012 (2); Pieper 2012 (12); Singh 2012 (3)
	1.2 Report methods used to present or assess RoB of primary studies from the included SRs and additional primary studies (and tool(s) used)	Becker & Oxman 2008 (8); Cooper 2012 (9); Hartling 2012 (1); JBI 2014 (10, 11); Pieper 2012 (12)
	1.3 Report methods used to deal with missing, flawed or discrepant RoB assessments of primary studies	Robinson 2015 (13-17)
	1.4 Report how RoB assessments of SRs and primary studies were incorporated into the interpretation of the results of the overview	Robinson 2015 (13-17); Hartling 2012 (1)
	1.5 Report the number of authors required to assess the RoB in SRs and primary studies, and any processes used to minimise bias and error in assessment	Robinson 2015 (13-17); Hartling 2012 (1)
**2.0 Synthesis, presentation, and summary of the findings**		
	2.1 Report methods for summarising the SR results	Cooper 2012 (9); Hartling 2012 (1); JBI 2014 (10, 11); Li 2012 (2); Singh 2012 (3)
	2.2 Report methods for quantitatively synthesizing the SR results	Cooper 2012 (9); Hartling 2012 (1); Smith 2011 (18)
	2.3 Report methods for dealing with additional studies in the synthesis	Inferred
	2.4 Report methods (non-statistical and statistical) for assessing reporting bias in SR and primary studies	Caird 2015 (19); Cooper 2012 (9); Hartling 2012 (1); JBI 2014 (10, 11); Singh 2012 (3); Smith 2011 (18)
	2.5 Report methods for dealing with and presenting overlap of primary studies across SRs	Inferred
	2.6 Report methods or the tool used to deal with and present discordant results, interpretations or conclusions of SRs	Inferred

**3.0 Assessment of the certainty of the evidence arising from the overview**	
3.1 Report methods used to assess certainty of the evidence arising from the overview (and the tool used)	Hartling 2012 (1); Li 2012 (2)
3.2 Report methods used to deal with missing, flawed or discrepant assessments of the certainty of the evidence extracted from SRs	Robinson 2015 (13-17)
3.3 Report methods used to deal with overlap in the primary studies contributing to assessments of the certainty of evidence extracted from SRs	Inferred
3.4 Report how certainty of the evidence assessments were incorporated into the interpretation of the results of the overview	Li 2012 (2)
3.5 Report the number of overview authors required to assess (or extract) the certainty of the evidence, and any processes used to minimise bias and error in assessment	Baker 2014 (20); Becker & Oxman 2008 (8); Cooper 2012 (9); JBI 2014 (10, 11); Li 2012 (2); Ryan 2005 (21)
**4.0 Overall**	
4.1 Report conflicts of interest, dual authorship, and funding sources	Büchter 2011 (22, 23)

JBI: Joanna Briggs Institute; RoB: risk of bias; RoBIS: Risk of Bias In Systematic reviews; SRs: systematic reviews.

References

1. Hartling L, Chisholm A, Thomson D, Dryden DM. A descriptive analysis of overviews of reviews published between 2000 and 2011. PloS one. 2012;7(11):e49667.
2. Li LM, Tian JT, Tian H, Sun R, Liu Y, Yang K. Quality and transparency of overviews of systematic reviews. J Evid-Based Med. 2012;5(3):166-73.
3. Singh JP. Development of the Metareview Assessment of Reporting Quality (MARQ) Checklist. Revista Facultad de Medicina de la Universidad Nacional de Colombia. 2012;60(4):325-32.
4. Chandler J, Churchill R, Higgins J, Lasserson T, Tovey D. Methodological standards for the conduct of new Cochrane Intervention Reviews. Sl: Cochrane Collaboration. 2013.
5. Higgins JPT, Green, S. (editors). . Cochrane Handbook for Systematic Reviews of Interventions Version 5.1.0 [updated March 2011]: The Cochrane Collaboration; 2011. Available from http://handbook.cochrane.org.
6. Liberati A, Altman DG, Tetzlaff J, Mulrow C, Gotzsche PC, Ioannidis JP. The PRISMA statement for reporting systematic reviews and meta-analyses of studies that evaluate health care interventions: explanation and elaboration. Annals of internal medicine. 2009;151.
7. Oxman AD, Guyatt GH. Validation of an index of the quality of review articles. Journal of clinical epidemiology [Internet]. 1991; 44(11):[1271-8 pp.]. Available from: http://onlinelibrary.wiley.com/o/cochrane/clcmr/articles/CMR-180/frame.html.
8. Becker LA, Oxman, A.D. Chapter 22: Overviews of reviews. In: Higgins JPT GSE, editor. Cochrane Handbook for Systematic Reviews of Interventions. Hoboken, NJ: John Wiley & Sons; 2008. p. 607-31.
9. Cooper H, Koenka AC. The overview of reviews: unique challenges and opportunities when research syntheses are the principal elements of new integrative scholarship. American Psychologist. 2012;67(6):446-62.
10. Aromataris E, Fernandez R, Godfrey CM, Holly C, Khalil H, Tungpunkom P. Summarizing systematic reviews: methodological development, conduct and reporting of an umbrella review approach. Int J Evid Based Healthc. 2015;13(3):132-40.
11. Joanna Briggs Institute. Methodology for JBI Umbrella Reviews. South Australia: The University of Adelaide; 2014.
12. Pieper D, Buechter R, Jerinic P, Eikermann M. Overviews of reviews often have limited rigor: a systematic review. Journal of clinical epidemiology. 2012;65(12):1267-73.
13. Robinson C, Halligan S, Taylor SA, Mallett S, Altman DG. CT colonography: a systematic review of standard of reporting for studies of computer-aided detection. Radiology. 2008;246(2):426-33.

14. Robinson KA, Chou R, Berkman ND, Newberry SJ, Fu R, Hartling L, et al. Twelve recommendations for integrating existing systematic reviews into new reviews: EPC guidance. Journal of clinical epidemiology. 2016;70:38-44.
15. Robinson KA, Chou, R., Berkman, N.D., Newberry, S.J., Fu, R., Hartling, L., Dryden D., Butler, M., Foisy, M., Anderson, J., Motu'apuaka, M.L., Relevo, R., Guise, J.M., Chang, S. Integrating Bodies of Evidence: Existing Systematic Reviews and Primary Studies. Rockville (MD): Agency for Healthcare Research and Quality (US); 2008.
16. White CM, Ip S, McPheeters MC, Tim S., Chou R, Lohr KN, et al. Using existing systematic reviews to replace de novo processes in conducting comparative effectiveness reviews. Rockville, MD: Agency for Healthcare Research and Quality; 2009.
17. Whitlock EP, Lin JS, Chou R, Shekelle P, Robinson KA. Using existing systematic reviews in complex systematic reviews. Annals of internal medicine [Internet]. 2008; 148(10):[776-82 pp.]. Available from: http://onlinelibrary.wiley.com/o/cochrane/clcmr/articles/CMR-14250/frame.html.
18. Smith V, Devane D, Begley CM, Clarke M. Methodology in conducting a systematic review of systematic reviews of healthcare interventions. BMC medical research methodology. 2011;11(1):15.
19. Caird J, Sutcliffe K, Kwan I, Dickson K, Thomas J. Mediating policy-relevant evidence at speed: are systematic reviews of systematic reviews a useful approach? Evidence & Policy: A Journal of Research, Debate and Practice. 2015;11(1):81-97.
20. Baker PRA, Costello JT, Dobbins M, Waters EB. The benefits and challenges of conducting an overview of systematic reviews in public health: a focus on physical activity. J Publ Health. 2014;36(3):517-21.
21. Ryan R, Hill, S., editor Developing an overview of systematic reviews: a framework for synthesising the evidence on interventions to improve communication. Cochrane Colloquium; 2005; Melbourne, Australia.
22. Büchter R, Pieper, D., Jerinic, P. Overviews of systematic reviews often do not assess methodological quality of included reviews. Poster. 19th Cochrane Colloquium;; 2011 Oct 19-22; Madrid, Spain: Cochrane Database Syst Rev; 2011. p. 105-6.
23. Büchter R, Pieper, D., editor How do authors of Cochrane Overviews deal with conflicts of interest relating to their own systematic reviews? Cochrane Colloquium; 2015; Vienna, Austria.
